# Supporting Muslim undergraduate medical students through medical school: lessons from a novel, student-led case-based learning intervention

**DOI:** 10.3389/fmed.2025.1545437

**Published:** 2025-05-09

**Authors:** Zain Mohammed, Hafsah Ba, Linta Nasim, Emily Roisin Reid

**Affiliations:** ^1^Faculty of Science, Engineering and Medicine, Warwick Medical School, University of Warwick, Coventry, West Midlands, United Kingdom; ^2^University Hospitals Coventry and Warwickshire NHS Trust, Coventry, United Kingdom

**Keywords:** Muslim medical students, cultural safety in medical education, equity diversity and inclusion (EDI), case-based learning (CBL), student-led faculty development, faith-based medical education interventions, inclusive medical education, religion and medical training

## Abstract

**Introduction:**

Muslim medical students in the UK face discrimination, microaggressions, and inadequate institutional support, affecting their well-being, academic experience and outcomes. Using Case-Based Learning (CBL) as a pedagogical framework, a novel student-led teaching intervention was created and delivered to small groups of faculty and students, with the aim of enhancing awareness, promoting inclusivity, and supporting educators of these issues.

**Methods:**

This CBL intervention was designed and led by Muslim medical student facilitators with subject expertise and previous experience in implementing curricular interventions. Scenarios based on real-life student experiences explored authentic challenges Muslim students face during their medical studies. Data were gathered to assess the effectiveness of the teaching innovation against its aims in the following formats: (1) in-session participant feedback, (2) transcriptions of the in-session discussions which demonstrated participant learning gain, and (3) notes from the post-session facilitator team reflections. These data were thematically analyzed using Braun and Clarke's six-point framework, with individuals coding the data individually and collectively across three meetings to refine and agree upon the themes.

**Results:**

Five key themes of insights emerged from the data: Staff and Student cultural literacy relating to Islam, Facilities and Environment, Curriculum, Policy and Processes, Islamophobia and discrimination. The in-session discussions evidenced that participants had increased their cultural literacy and awareness of Muslim students' needs and identified practical solutions, including inclusive scheduling, making appropriate prayer facilities available to enable equitable educational attainment, providing clear clinical attire guidelines, and providing robust reporting mechanisms. The facilitators reflected that the students-as-experts aspect of the intervention equalized the usual faculty-student power dynamics. This promoted a sense of partnership that enabled participants in the sessions to take ownership of their own learning.

**Discussion:**

CBL presented a valuable format for student-faculty discussions to promote cultural competence and equity in medical education. Variability in assumed knowledge and cultural literacy posed challenges, reinforcing the need for broader implementation of Equity, Diversity and Inclusivity (EDI) training and enhanced institutional support networks to develop cultural literacy further.

**Conclusion:**

This student-led CBL educational innovation brokered a dialogue between students and faculty around solutions to the challenges faced by Muslim medical students. Given its success, student-led staff training could be expanded to address challenges faced by other minority groups, ensuring a more equitable and culturally competent learning environment.

## 1 Background and rationale

Muslims constitute 10% of the medical workforce but face disproportionate challenges in training and career progression (1, 2). The General Medical Council (GMC) reports that Muslim trainees have the lowest success in their Annual Review of Competence Progression assessments and lower postgraduate examination pass rates compared to peers (3). Furthermore, minoritized ethnic heritage staff comprise 42% of the medical workforce but hold only 11.2% of very senior leadership roles (4). These disparities stem from systemic barriers, including bias in promotion decisions (5, 6) and limited access to professional networks and mentorship (7). Other factors include inconsistent institutional support for religious practices such as prayer and dress codes; policy regarding hijab in theater or “bare below the elbows” (8); and feelings of “othering” due to implicit or explicit bias (5, 9, 10). Such systemic barriers limit career progression and can directly impact patient care and public health. A diverse and representative medical workforce is crucial for ensuring culturally competent and equitable healthcare, particularly in the UK's increasingly diverse patient population (6). These challenges have transcended national boundaries, as one United States study identified that Muslim nursing students faced similar challenges (11).

This study sought to evaluate the role of educational interventions aimed at medical undergraduate educators and faculty in tackling these barriers through “Case-based learning” (CBL). This approach employs an enquiry-based learning model, enabling participants to explore new concepts, such as Islamic practices, while building on existing knowledge, emphasizing the role of empathy and solution building. Studies have demonstrated that scenario-based approaches facilitate perspective-taking, problem-solving, and engagement with real-world complexities. CBL effectively addresses racial and cultural inequalities and has been utilized across disciplines beyond medical training (12–16).

Given that many of these systemic barriers emerge early within medical training, targeting undergraduate education through a structured education intervention offers a proactive opportunity to address these challenges. Current research on Muslim undergraduate experience focused on broad themes of diversity and differential experience of minorities (17, 18), the impact of counter-terror measures and surveillance (19), Islamophobia at university (20) and limited appropriate emotional and psychological support services (21). Although this is valuable and pertinent to recognizing the challenges encountered by Muslim medical students in the UK, existing research does not evaluate interventions to mitigate the structural, professional and cultural barriers Muslim medical students face. A single study describes the success of a “Muslim Student Guide to Medical School” companion for new undergraduates. This intervention was effective in empowering students directly, rather than addressing the broader structural and institutional barriers that contribute to these challenges (22).

Addressing these barriers requires more than individual adaptation; rather, it demands a shift in the way cultural understanding and inclusion are approached within medical education, highlighting the importance of moving beyond “cultural competence”. Historically, “cultural competence” has been the dominant framework used in medical education to prepare healthcare professionals for interaction with patients from diverse backgrounds (23, 24). It requires healthcare providers to understand the cultural norms and values of a patient group. However, this approach has faced criticism for promoting a superficial, checklist-based approach that emphasizes cultural knowledge acquisition over meaningful reflection and systemic change (23–25). Recognizing that culture plays a significant role in patient care and the integration of colleagues within the healthcare workforce (26), “cultural safety” has emerged as a more comprehensive framework. Cultural safety recommends healthcare professionals and institutions engage in continuous self-reflection, address structural biases, and deliver care that is defined as safe by the patient's experience (25). In contrast to “cultural competence”, “cultural safety” emphasizes the importance of awareness of personal limitations and power dynamics, avoidance of assumptions, and the need for healthcare providers to engage in continuous self-evaluation.

CBL aligns closely with the principles of “cultural safety” by encouraging reflective practice, often in groups and critical examination of bias. It provides a structured environment for exploring how personal and institutional biases influence healthcare delivery (27). By facilitating open discussion on issues of religious and cultural identity, CBL supports deeper understanding and meaningful dialogue. This approach allows participants to identify and address the structural barriers that contribute to disparities in healthcare delivery and career progression. Equity, Diversity, and Inclusion (EDI) training, conducted through simulation teaching, has enhanced participants' knowledge, insight, self-efficacy, and EDI-related competence (28). Warwick Medical School has effectively implemented a CBL approach for student-led EDI training of medical faculty (29). Thus, using CBL was a natural choice to introduce concepts of cultural safety, humility, and sensitivity, addressing Muslim medical students' specific needs and challenges. The novelty of this activity lies in the student-led CBL format, whereby the expertise of student facilitators stems from lived experience, which may not be present among participants and helps to inform and guide the activity.

## 2 Aims for this educational innovation

**Figure 1:**
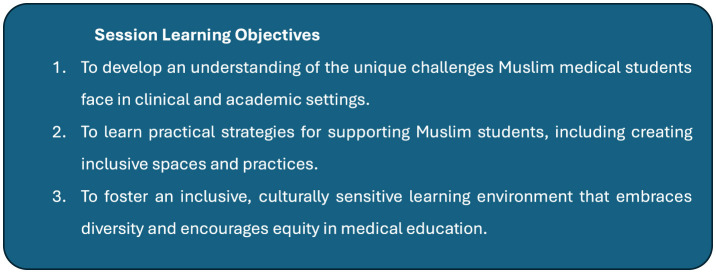
Session learning objectives.

This study sought to answer the question: “What key insights emerged from a student-led CBL intervention designed to enhance participants' understanding of the challenges faced by Muslim medical students and improve their ability to identify personal and institutional strategies for addressing these challenges?” Specific learning objectives for the session have been outlined in Figure 1.

This intervention aimed to develop participants' cultural awareness and humility, improve understanding of the Islamic faith and doctrines and enable reflection on how institutional and personal biases impact Muslim medical students' educational experiences and outcomes. Through a student-led CBL format, the intervention sought to foster practical, solution-focused dialogue and drive institutional change. In doing so, this study seeks to bridge the gap in evidence by highlighting how this student-led CBL training may be helpful for faculty and students as an intervention to promote cultural awareness and inclusivity within medical education.

## 3 Theoretical frameworks, principles, and standards

During CBL, learners are presented with real-world scenarios or clinical cases, identifying key issues, exploring underlying concepts and vocabulary, and proposing solutions through peer discussion and investigation. The process typically unfolds in three phases: learners first identify gaps in their knowledge, then explore the case collaboratively, discuss potential solutions and underlying biases, and finally present conclusions. Learners should reflect on the process and receive feedback to support continuous improvement (12). In this instance, a peer-led format creates a psychologically safe environment, encouraging open dialogue and exploration of complex social and cultural issues.

This process is underpinned by established pedagogical theories, incorporating a constructivist approach that emphasizes knowledge construction through experiences and social interactions. Constructivism requires that learners develop a deeper understanding to engage with real-world problems, reflect on existing knowledge, and develop their understanding through peer discussion and guided inquiry (30, 31). Research has shown that CBL enhances clinical reasoning, reflective practice, and learner engagement, with participants reporting increased motivation and deeper understanding. Educators similarly value CBL for its ability to foster independent thinking and active participation (20).

The use of CBL aligns with the professional competencies outlined by the General Medical Council (GMC) in Good Medical Practice (2024), through reflective practice, and effective communication. The GMC emphasizes the importance of treating patients and colleagues with respect, recognizing the impact of personal and systemic biases, and fostering an inclusive learning and clinical environment (32).

CBL provides a structured framework for developing these competencies, encouraging participants to reflect on their assumptions and engage with diverse perspectives. Through peer-led inquiry, participants are challenged to identify practical solutions for addressing institutional barriers and improving cultural safety within healthcare settings. Developing these competencies is essential for delivering equitable and culturally competent care within the NHS.

CBL's peer-led format makes it effective for exploring sensitive issues related to religious identity and discrimination. Drawing on the lived experiences of Muslim medical students, the authors designed a case study that facilitated discussions to achieve key learning outcomes. CBL's ability to engage learners with real-world complexities allowed participants to explore how systemic bias and institutional barriers affect Muslim medical students' educational and professional experiences and outcomes. By fostering reflective practice and encouraging solution-focused dialogue, the intervention aimed to improve understanding of cultural identity and equip participants with strategies to address both personal and institutional biases. This intervention demonstrated that CBL was an effective approach to exploring concepts of cultural safety, humility, and addressing the specific needs of Muslim medical students and challenges.

## 4 Methodology

### 4.1 Intervention design

This CBL intervention was designed and led by Muslim medical student facilitators with subject expertise and previous experience in implementing curricular interventions. The authors created scenarios based on real-life student experiences of the kinds of barriers explored in the cases. The students had previous experience in facilitating sessions, in addition to lived experience and subject expertise. They chose CBL as a common method that is familiar within medical education, to facilitate discussions and explore authentic challenges Muslim students face during their medical studies.

Sessions were delivered across three settings between December 2023 and July 2024:

- A senior faculty event at Warwick Medical School (*n* = 45; including those who viewed the recording);
- A clinical medical undergraduate CBL facilitator training session in February 2024 (*n* = 24);
- A workshop at the ASME Annual Conference in July 2024, attended by 44 medical educators and students.

**Figure 2:**
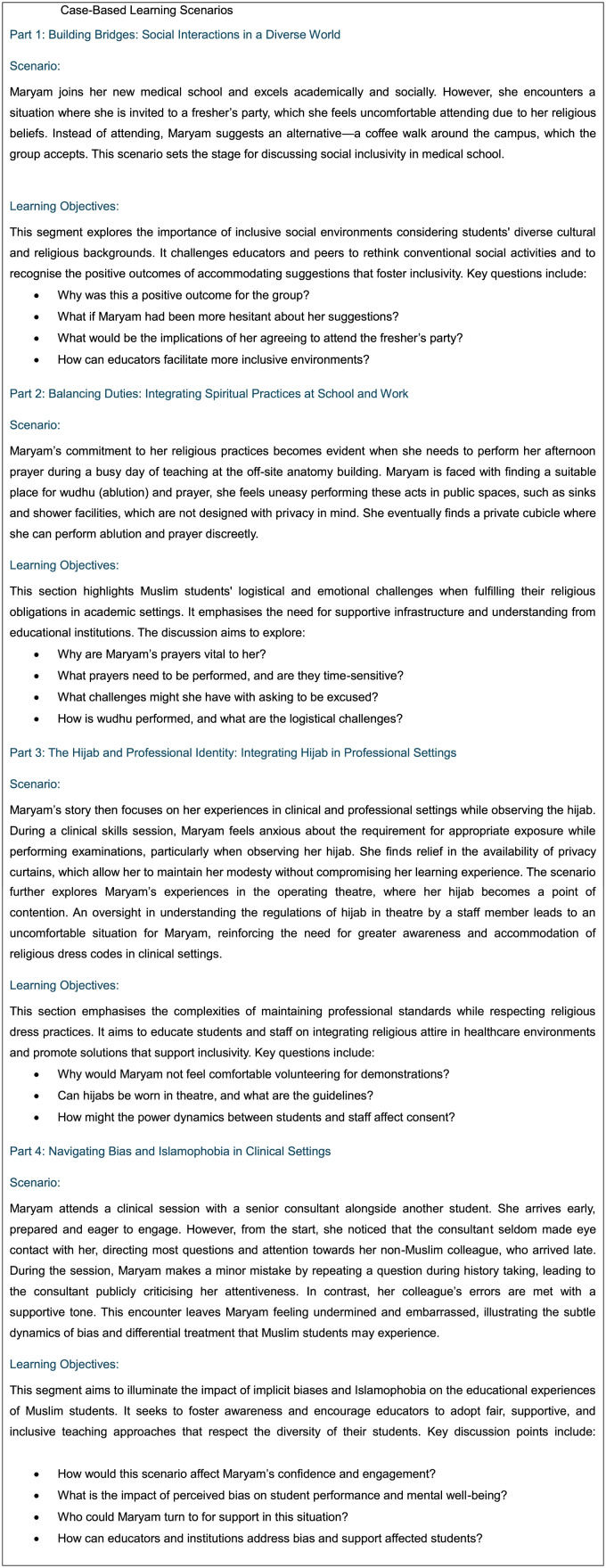
Case-based learning scenarios.

The sessions applied core principles of CBL, incorporating co-designed scenarios based on lived experience. Each scenario was followed by clarification of unfamiliar terms, small-group discussion, and collective solution-building. Scenarios focused on four key domains: navigating social norms, religious practices in clinical education, modesty and the hijab, and experiences of Islamophobia. Narratives are presented in Figure 2.

Participants were recruited to attend the sessions using purposive sampling to capture a wide range of perspectives. Attendees included medical students, faculty, tutors, course directors, and clinical staff, from both Muslim and non-Muslim backgrounds, with a range of ethnicities and levels of seniority. Participation was voluntary, and no exclusion criteria were applied. The initial two sessions for medical school faculty and CBL facilitators invited participants via internal email, whilst the conference session was open to all attendees of the conference and advertised on the itinerary. All sessions created safe discussion spaces, guided by trained facilitators with lived experience. Participants were grouped into 6–10 individuals, discussed scenarios with facilitators guiding conversations and later reconvened to share insights with the larger group The varying settings influenced participant engagement: small group classroom-style sessions encouraged richer interaction.

The participants consented to sharing their data for the purposes of evaluation and wider dissemination. As this article constitutes a secondary analysis of existing anonymous information, ethical approval was not required.

### 4.2 Data collection and analysis

The evaluation wished to move beyond a simple participant satisfaction, and to achieve higher levels of Kirkpatrick's framework (33), capturing evidence of learning and intention to change behavior and practice. Therefore, to evaluate the effectiveness of the teaching innovation against its aims, we gathered and grouped the following data:

- Facilitator annotations of solicited participant feedback at the end of each session, cross-referenced with transcriptions of recorded content.
- Transcriptions and annotations from the in-session discussions, with a focus on participant learning gain (i.e. the difference in where they started in discussions to where they ended up in their understanding).
- Facilitator reflections, collated in debriefing meetings following each event.

Data were analyzed using thematic analysis (34). Coding was performed independently by multiple facilitators, who later met for three analytical group discussions to refine themes, resolve discrepancies, and agree on the final themes. The collaborative thematic cross-checking was employed to mitigate bias and ensure rigor across the sources of data.

## 5 Results

### 5.1 Session evaluation and thematic analysis

At the end of the sessions, the facilitators sought feedback into the session's effectiveness against it's stated aims. Participants consistently praised the respectful and inclusive environment created during the session, noting the sensitivity of facilitators. Comments about the “thoughtful, inclusive, and kind leading of this session” underscored the positive atmosphere, as well as highlighting the high value staff portrayed toward the student facilitators. The session was recognized for its educational impact, particularly in increasing awareness of Islamic practices and the challenges faced by Muslim students. Attendees also appreciated the practical elements of the session, including the use of the CBL approach and visual aids, which effectively engaged participants. Based on participant feedback from the first iteration, the session was refined to enhance engagement and learning. The number of slides was reduced, allowing for more interactive discussions and real-life case scenarios, while smaller group discussions encouraged deeper dialogue. To support continued learning, take-home resources were introduced, including handouts, glossaries of key Islamic terms, prayer schedules, and guidance on religious accommodations in medical settings. These improvements enhanced interactivity, inclusivity, and practical relevance, ensuring a more impactful learning experience.

**Figure 3:**
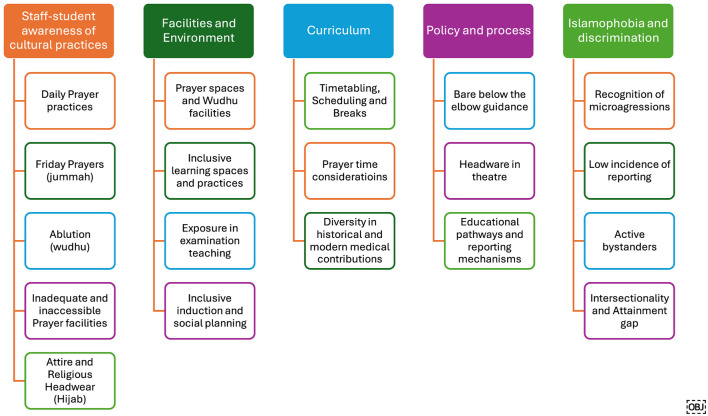
Themes and subthemes developed through Muslim medical student experience CBL.

Five overarching themes with subthemes emerged from the data (see Figure 3). These represent the key areas that the CBL intervention demonstrated the most impact relating to participants' learning, both in terms of challenges faced by Muslim medical students and enablers they identified they could implement to improve their student experience going forwards.

### 5.2 Staff-student awareness of cultural practices

Participants demonstrated limited awareness of prayer room requirements, Islamic prayer times, their seasonal variations, and the implications of missing prayers. Muslim prayers are performed at specific times dictated by the sun's position, such as Dhuhr (midday) and Asr (afternoon), each within defined periods. Participants also had limited awareness regarding the significance of Jummah, the Friday congregational prayer performed in a larger assembly. Missing normal prayer times requires later compensation, which is less than ideal for Muslim students. This lack of understanding can lead to scheduling issues that mean students miss learning opportunities, resulting in cumulative deficits to their learning outcomes, as well as unintentional insensitivity toward Muslim students' needs. The variability of Muslim prayer times presents challenges in aligning with standard academic schedules. The discussions within the sessions explored that whilst tailoring timetables to specific prayer times may not be feasible, providing designated periods and appropriate facilities for prayer and wudhu is essential. Some institutions have adopted solutions such as “protected lunch breaks”; time that is safeguarded from any educational activities for students.

Similarly, the concept and practice of wudhu (ablution) were unfamiliar to many participants, including its frequency (up to five times daily) and requirements, such as washing the hands, face, arms, head, and feet, typically taking about 5 min. This knowledge gap results in insufficient facilities and inadequate support for Muslim students to meet their religious obligations. For example, the lack of private spaces for hijab removal, interruptions during wudhu often in shared spaces, and the impracticality of washing feet in sinks, which poses barriers to performing wudhu with dignity, can negatively impact students' learning experience. Accessible washing, prayer spaces, and regular rest intervals can accommodate students' religious practices without significantly modifying timetables and schedules. While some UK universities and schools provide wudhu-specific facilities, their availability is inconsistent, leaving many students reliant on public or private toilets, which are less ideal.

### 5.3 Facilities and environment

The scenario presented in this case was derived directly from students' real-life experiences in medical school, prompting participants to reflect on their perspectives. There was unanimous agreement that requiring students to perform examinations on one another in an open setting was unacceptable. Privacy measures, such as curtains, were identified as essential to ensure comfort and dignity, benefiting all students alike.

The discussion also raised concerns about students' expectations to examine each other. Participants noted that students might feel pressured to comply, even if uncomfortable, to support their peers' learning. As a solution, using simulated patients or actors in teaching sessions involving physical examinations was proposed as a more inclusive and suitable alternative for medical schools to adopt.

## 6 Curriculum

### 6.1 Scheduling, breaks, and prayer times

Long teaching sessions without breaks posed challenges for students needing to perform prayers, particularly during winter when prayer times are closely spaced. Participants recommended incorporating brief 5–10-min breaks every 45–50 min to accommodate prayer needs. This adjustment would enable Muslim students to fulfil their obligations without disrupting the session's flow while also benefiting all students by providing rest periods. Such an approach promotes inclusivity and respects the diverse needs of the student body.

### 6.2 Policy and process

#### 6.2.1 Inconsistent policies on bare below the elbows

Discussions highlighted significant inconsistencies in applying bare-below-the-elbows policies across various medical schools and NHS trusts. According to NHS guidance, long sleeves may be worn when staffs are not in direct contact with patients, and disposable sleeves can be used over clothing when necessary (35). However, participants reported variability in how these policies are enforced, often leading to confusion and conflicting instructions from colleagues. In some cases, students shared that they had been reprimanded for wearing long sleeves despite the guidance, further exacerbating their challenges. These inconsistencies underscore the need for clearer communication and uniform enforcement of policies to ensure fairness and understanding across clinical settings.

#### 6.2.2 Awareness of guidelines for religious headwear in theater

Participants demonstrated limited awareness of the rules and regulations regarding religious headwear in theater. Many were unfamiliar with the 2020 uniforms and workwear guidance for NHS employers, which permits the use of normal cloth headscarves during theater attendance, provided they are subsequently washed at 60°c, with or without an additional theater cap (35). Facilitators played a key role in highlighting this guidance, underscoring the importance of raising awareness about diversity in workwear policies across NHS trusts.

#### 6.2.3 Educational pathways and reporting mechanisms

There was differential knowledge from the participants of reporting processes and when to advise students on escalation. It is essential that Muslim students know where to seek support and feel confident reporting concerns without fear of reprisal or minimization.

### 6.3 Islamophobia and discrimination

#### 6.3.1 Recognition of islamophobia and microaggressions

The case study highlighted instances based on real-life Muslim students' experiences in which wearing visible religious symbols such as the hijab has resulted in Islamophobia and microaggressions, both explicit and implicit. These biases may manifest as differential treatment, disproportionate criticism, or exclusion from educational interactions, as seen in Maryam's case. There was an awareness that such behaviors create an unequal learning environment where Muslim students may feel unwelcome or unfairly scrutinized, when contrasted to the lack of knowledge regarding procedures on bare below the elbows or religious headwear, which can manifest as implicit discrimination,

#### 6.3.2 Low likelihood of reporting singular events

Participants discussed different levels of understanding around how students are often hesitant to report isolated incidents of bias or Islamophobia, fearing these may be dismissed as minor or unintentional. There was an appreciation that this reluctance leaves recurring issues unaddressed, as individual events may only appear significant when viewed collectively. Educators and institutions must emphasize the importance of reporting even singular incidents, as they contribute to patterns of discriminatory behavior. Clear and accessible reporting pathways, combined with normalizing their use, can empower students to address these experiences.

#### 6.3.3 The importance of active bystanders

There was a good understanding among participants that creating a culture where peers and educators act as active bystanders will address Islamophobia and discrimination in real-time. Discussions around the cases showed that participants were generally aware that recognizing and intervening in subtle instances of bias can prevent such behaviors from continuing unchecked, with examples demonstrating how bystander student training has made positive inroads to developing a culture of respect and accountability in clinical education settings, promoting inclusivity and reducing the risk of harm to students like Maryam.

#### 6.3.4 Intersectionality and the attainment gap

The session underscored how intersecting identities, such as race, gender, and religion, can amplify biases. For Maryam, being both a Muslim and a woman heightens her vulnerability to implicit biases in clinical settings. These compounded biases contribute to the attainment gap observed among minority students, negatively affecting their learning experiences and subsequent academic performance. The ensuing discussion amongst participants raised a level of understanding and awareness of the importance of addressing intersectionality, which is vital to creating equitable educational environments.

## 7 Discussion

This study offers significant insights into enhancing cultural awareness and humility among faculty in medical education through a student-led, faith-based CBL intervention. It demonstrates how lived experiences, when presented through a structured educational partnership intervention, can foster deeper empathy, stimulate institutional reflection, and encourage practical strategies for inclusion.

The findings of this study reinforce a growing body of literature supporting the use of CBL as a pedagogical tool to address EDI in health professions education. CBL facilitates structured, experiential learning and promotes critical reflection in safe environments, particularly when grounded in real-life scenarios involving marginalized learners (36). This intervention contributes further evidence that CBL can be successfully adapted for faculty development, particularly when the content is co-designed with students and rooted in lived experience.

The study also supports existing research highlighting the potential for student-led educational initiatives to inform and reshape institutional practice. Prior work by Nazar et al. (37) underscores the value of student-led diversity education in decolonizing curricula, revealing how learners can expose epistemic biases and prompt institutional reform. Similarly, Mind the Gap (38) exemplifies how student-initiated resources can challenge clinical teaching norms and promote more inclusive, representative practice. These interventions, like the one evaluated here, highlight how students—when positioned as co-educators—can drive pedagogical innovation and contribute to the creation of culturally safe learning environments.

By placing students in facilitative roles and using authentic case studies, this model challenges traditional hierarchies and repositions students as educators—an approach mirrored by Gallier-Birt et al. (39) and Warnock et al. (40), who found that student-led EDI training fosters dialogue, reflection, and meaningful changes in staff awareness and behaviors. This study strengthens that evidence base, demonstrating how such peer-to-staff training can be scaled within formal medical school structures. Similarly, Davis et al. (14) describe a student–staff partnership at Warwick Medical School to revise and decolonize CBL case content, illustrating how learners can drive curriculum reform by integrating perspectives historically excluded from mainstream medical narratives.

Moreover, this intervention addresses a critical gap in the literature around structured, faith-sensitive approaches to support Muslim medical students. While previous efforts, such as the Manchester Muslim Medical Student Guide (22), have highlighted key challenges and provided valuable resources, few educational interventions have translated this knowledge into faculty-facing training. This study offers a novel model that not only shares lived experiences but uses them to drive institutional action, aligning with wider calls to integrate cultural and religious competence into EDI frameworks (28, 41).

The intervention aligns with broader inclusive curriculum frameworks that advocate for pedagogical reform through co-production, cultural humility, and systemic change. Lokugamage et al. (42) illustrate how student–staff partnerships can drive meaningful curricular reform in medical education, using case-based methods to center cultural safety, epistemic plurality, and learner voice. These principles are echoed in the Inclusive Higher Education Framework (43), which outlines key domains for advancing inclusive teaching practice. These include structures and processes, curriculum design and delivery, and community and belonging. All of these were addressed through the collaborative nature and objectives of this intervention.

### 7.1 Implications for practice

This intervention was shown to be effective in meeting its aims of improving understanding and cultural literacy relating to the barriers experienced by Muslim students. The implications for practice are summarized in Table 1, drawing together the barriers identified during the sessions as well as enablers which were identified.

**Table 1:** Topics explored and solutions proposed from the CBL sessions to the challenges faced by Muslim medical students.

**Challenge**	**Explored topics**	**Proposed solution (CBL outcome)**
Socialization	Discussions centered on discomfort in alcohol-related social settings and strategies to create inclusive environments. Recognizing that such discomfort is not unique to Muslim students, the sessions emphasized the importance of accommodating diverse social preferences.	Supporting the growth of a Muslim student body that can advocate for itself and provide mentorship and guidance to other Muslim students. Having visible role models and a support community can empower students to navigate challenging environments. Establishing partnerships with organizations such as BIMA and university chaplaincies, which offer structured support for Muslim students and help address their unique needs within clinical and academic settings. Additionally, establishing a within-cohort Muslim student body would help provide students with familiarity and safe spaces to discuss concerns. Reported success with these bodies and faculty has resulted in the formulation of “Muslim Student Guidebooks”, EDI training and community cohesion projects.
Limited prayer/Wudhu space availability	The necessity of accommodating students' needs for performing ablution (wudhu) and prayers was discussed, emphasizing the provision of appropriate facilities and break times.	Establish dedicated, well-equipped prayer rooms on campuses and hospitals.
Prayer logistics and scheduling conflicts for Jummah (Friday) prayer	The challenges of balancing prayer times with a demanding medical curriculum were explored, considering the variability of prayer times due to seasonal changes. Solutions included timetable adjustments and ensuring accessible prayer spaces.	Implement protected or flexible lunch breaks to allow attendance. Encouraging faculty to work with chaplaincy services. This can benefit students of all faiths, promoting a more inclusive teaching schedule.
Lack of faculty awareness of worship needs	Encouraging clinicians to recognize and support the religious needs of incoming doctors was emphasized, promoting the inclusion of religious considerations in onboarding processes.	Embed faith-based inclusivity training in EDI programs. Comprehensive Equity, Diversity, and Inclusion (EDI) training is a key strategy for reducing bias in clinical settings. Such training should address cultural humility, unconscious bias, and the impact of microaggressions. Additionally, it must consider the intersectionality of identity factors to show how overlapping biases can intensify their effects. Clear communication within clinical teams, particularly during handovers, should be made to accommodate the need for prayer breaks. This practice fosters a supportive environment while ensuring that patient care remained unaffected. There is currently a gap in Supporting Trainees Entering Practice (STEP) forms, which currently lack specific questions about religious requirements. Including such considerations in these forms could help address the holistic well-being of medical students and professionals. These findings emphasize the need for greater awareness and proactive measures to accommodate the religious practices of Muslim medical students, fostering an inclusive and supportive learning environment.
Inconsistent NHS “Bare Below the Elbows” policy		Standardize policy enforcement across NHS Trusts and ensure faculty awareness through training.
Microaggressions and islamophobia in clinical settings		Introduce Active Bystander Training and strengthen reporting systems for discrimination.
Students hesitant to request accommodations	Encouraging clinicians to recognize and support the religious needs of incoming doctors was emphasized, promoting the inclusion of religious considerations in onboarding processes.	Normalize faith-based discussions in medical education and establish clear institutional policies. Inclusion of religious needs into STEP forms for newly qualified professionals.
Modesty concerns in clinical skills session	The appropriateness of mixed-gender sessions and maintaining dignity during examinations were addressed, advocating for options that respect students' cultural and religious beliefs. Modesty in clinical skills sessions and appropriate attire in sterile environments were examined, highlighting the need for guidelines that respect religious practices while maintaining professional standards. The specific requirements for maintaining aseptic conditions while respecting religious dress codes were discussed, leading to recommendations for accommodating head coverings in surgical settings.	Provide curtains for privacy and use simulated patients for examinations requiring exposure.
Unclear Policies on Religious Attire in Theater		Raise awareness of NHS uniform policies (2020) and ensure consistent implementation across NHS Trusts. Raising awareness through training and publicity.
Lack of transparent and effective reporting mechanisms for discrimination	Issues of Islamophobia in clinical environments were highlighted, underscoring the importance of reporting systems and the role of active bystanders in addressing discrimination.	Develop structured, well-publicized reporting mechanisms for students to report discrimination without fear of reprisal. Alongside fostering awareness and encouraging diverse representation, visible and effective reporting mechanisms are essential for addressing bias in clinical settings (33). Muslim students and other minorities must feel empowered that when they report instances of discrimination or prejudice, their concerns will be taken seriously and addressed promptly. Establishing transparent protocols for reporting, with clear, visible consequences for discriminatory actions, is critical for cultivating trust within medical education. When students see that reported incidents result in fair and consistent action, it can foster a safer and more inclusive learning environment. In addition, providing regular feedback on reported outcomes and processes—without compromising confidentiality—could further strengthen students' confidence in these systems. By making reporting mechanisms more accessible and maintaining transparency in response procedures, institutions can demonstrate their commitment to upholding equity, diversity, and inclusivity within medical training environments.
Limited awareness of diverse contributions to historical and modern medicine		Incorporate historical and contemporary contributions of diverse medical pioneers, including Muslim scholars like Ibn Sina (Avicenna) and Al-Zahrawi (Abulcasis), into medical curricula may help students and educators appreciate the diversity of thought that shapes contemporary medical practice and can foster a more inclusive educational environment (14, 43). Opportunities in elective modules.

The overwhelmingly positive feedback and actionable outcomes reported by participants affirm the intervention's effectiveness, and attest to the wider use and exploration of student partnership models. Specifically, this intervention supports the use of CBL to deliver sensitive, lived-experience-based content that promotes inclusion, empathy, and structural awareness. It provides a scalable model that can be adapted to other marginalized groups. Medical schools are encouraged to embed such interventions into faculty development programs, with formal recognition of student co-leads.

This intervention also reflects that institutional support is vital. Frameworks alone—such as those from the MSC and Advance HE—are insufficient without mechanisms to operationalize them, and support from individuals to overcome barriers that exist within educational systems. The results suggest that tapping into the deep expertise and lived experience that students possess, whilst offering them support to facilitate in safe, dialogical environments, is one such mechanism.

## 8 Conclusions and recommendations

This study demonstrates that student-led, case-based learning (CBL) can effectively enhance faculty understanding of the unique challenges faced by Muslim medical students. By centering lived experience and facilitating structured, dialogic learning, the intervention promoted cultural humility, disrupted hierarchical dynamics, and supported the co-creation of actionable strategies for institutional change. The overwhelmingly positive feedback and engagement from participants affirm the value of this approach in promoting inclusive educational practice and advancing equity within healthcare training.

**Figure 4:**
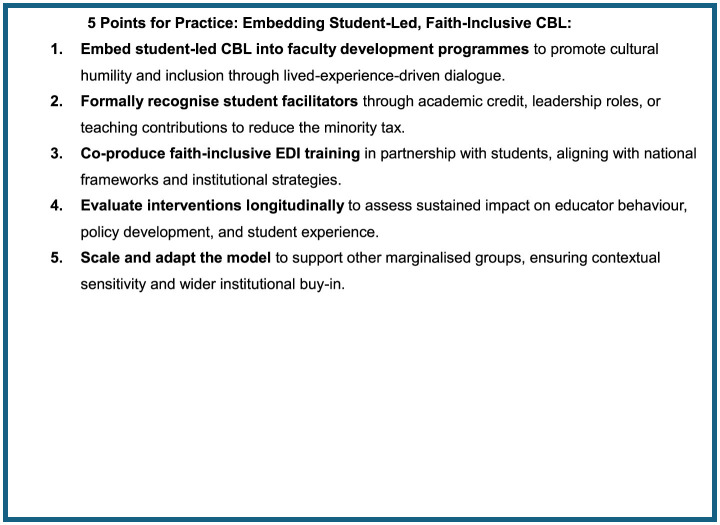
5 points for practice: embedding student-led, faith-inclusive CBL.

In light of these findings, we propose “5 Points for Practice” in Figure 4. Medical schools should consider integrating student-led CBL into faculty development programs, formally recognizing student facilitators and embedding such interventions within broader EDI strategies. National bodies such as the Medical Schools Council in the UK are well-placed to support the development of co-produced, faith-inclusive training guidance. Future research should evaluate the long-term impact of these interventions on educator behavior and policy implementation, assess scalability across institutions and marginalized groups, and incorporate independent or participant-led analysis to enhance methodological rigor. Adopting such inclusive, experiential learning models is a critical step toward fostering culturally safe and equitable medical education environments.

**Figure 5:**
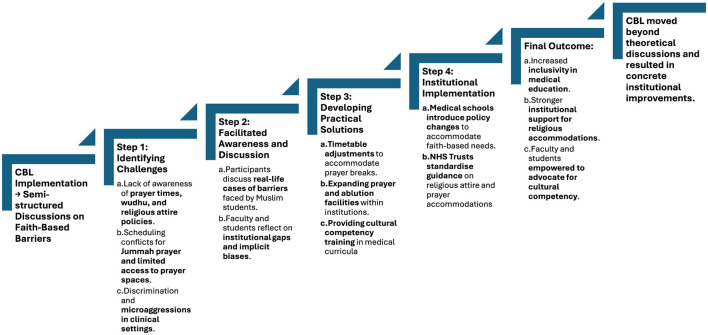
Flowchart of case-based learning (CBL) as an intervention for faith-based inclusivity in medical education. Challenges and solutions are explored in further detail in Table.

Since the interventions, the authors have seen a series of actions take place. Figure 5 presents a potential pathway for how educational changes follow as a result of the interventions that have taken place. A clear sequence of impact was observed, beginning with facilitated reflection, progressing through solution development, and culminating in institutional policy action. This stepwise process shows how student-led staff training is able to transition reflective dialogue to actionable outcomes—reinforcing CBL's role as a tool for both pedagogy and organizational transformation (14, 39).

### 8.1 Strengths

This study is the first of its kind to develop and evaluate a student-led, faith-based CBL initiative addressing the needs of Muslim medical students. Strengths include a scalable, replicable model of co-produced faculty development, alignment with national policy frameworks (e.g., MSC, UUK, Advance HE), and direct impact on educator practice. Notably, it was co-designed and facilitated by Muslim medical students with lived experience, positioning them as experts and allowing for the exploration of nuanced, real-world challenges often absent in traditional staff-led sessions. This student-led approach helped to rebalance faculty–student power dynamics and fostered a psychologically safe environment for open dialogue. The study employed a triangulated approach through multiple qualitative data sources that enabled a rich thematic analysis that strengthened the credibility and depth of the findings. The evaluation moved beyond simple participant satisfaction, reflecting a higher level of Kirkpatrick's framework (33) by capturing evidence of learning and intention to change practice, not merely feedback. Finally, the scalability of the intervention format was foregrounded through the deliberate choice of case-based learning (CBL), a pedagogical model familiar to both students and faculty in medical education, allowing potential application in different contexts internationally.

### 8.2 Limitations

The voluntary nature of participation likely introduced selection bias, with attendees predisposed to EDI engagement. The largest session drew from a single institution, limiting generalizability, although national conference participation mitigated this somewhat. Feedback was mostly oral and delivered in group settings, introducing potential social desirability bias. Thematic analysis was conducted by facilitators who also delivered the sessions, raising the risk of interpretive bias, albeit this was mitigated by following the Braun and Clarke framework for quality assurance (34). No formal follow-up was conducted to assess sustained impact or behavioral change. Future research should include longitudinal follow-up and involve institutional leaders to translate discussion into systemic change. Facilitator training should also address managing power dynamics in group settings to ensure equitable participation.

## Data Availability

The participants of this study did not give written consent for their data to be shared publicly, so due to the sensitive nature of the research supporting data is not available.
