# Supplementary material for: Supporting Muslim undergraduate medical students through medical school: lessons from a novel, student-led case-based learning intervention
Source: Front Med (Lausanne). 2025 May 9;12:1545437. doi: 10.3389/fmed.2025.1545437 (PMC12098333; doi:10.3389/fmed.2025.1545437)

SUPPORTING MUSLIM MEDICAL STUDENTS

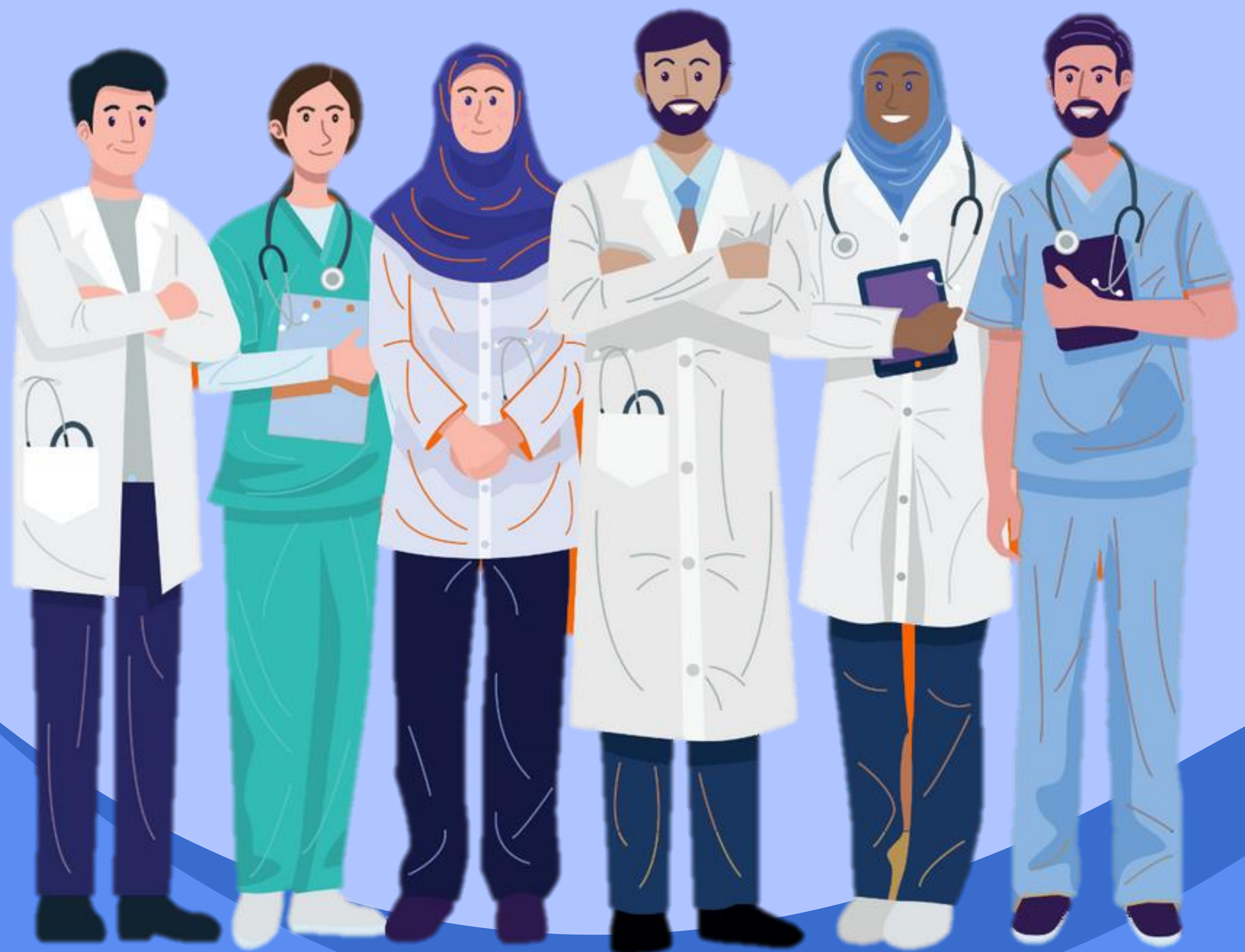

What educators need to know

Hafsah Ba | Linta Nasim | Dr Zain Mohammed

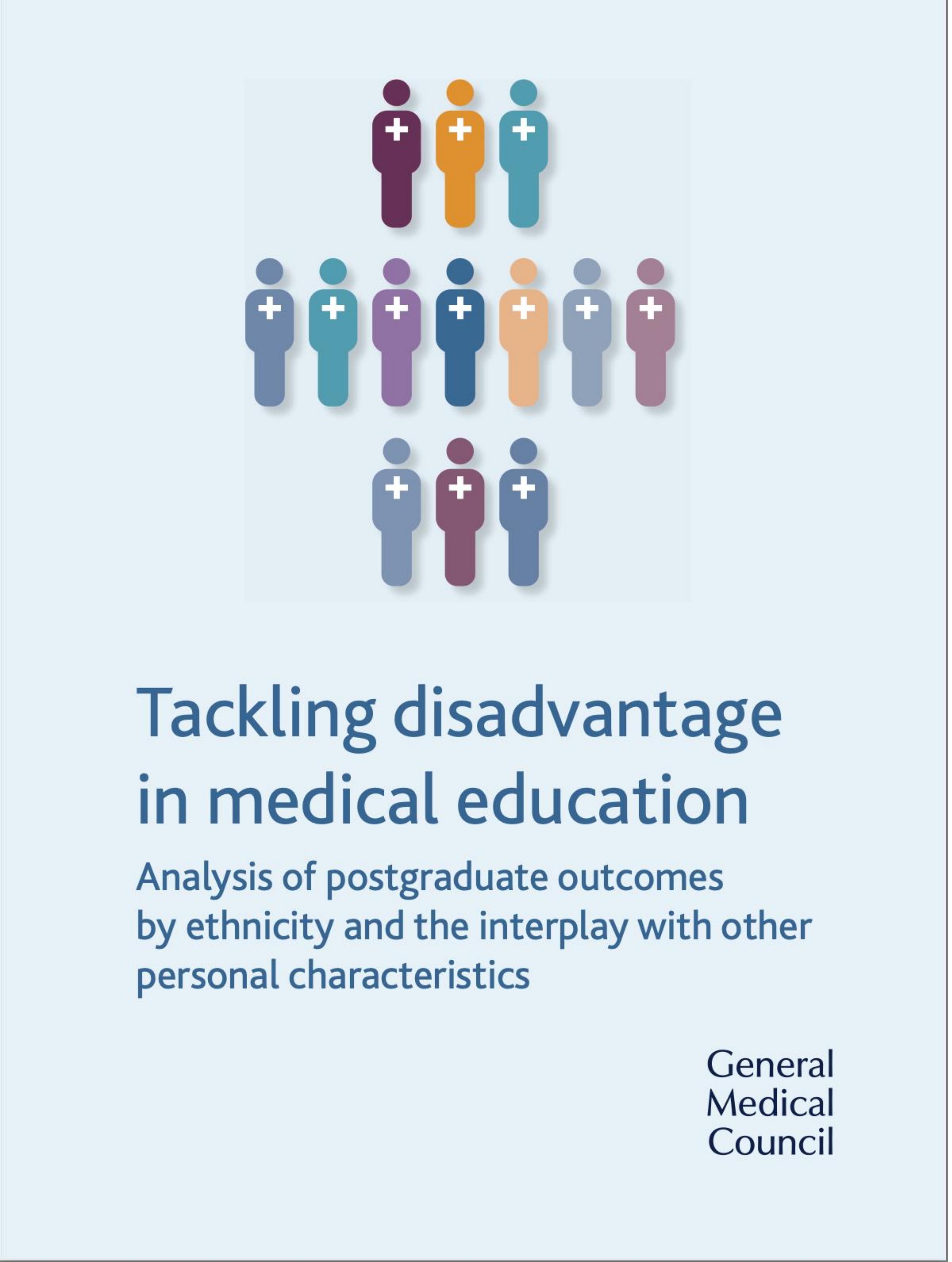

- Muslim trainees have the smallest proportion of successful outcomes at ARCP
- Muslim trainees have lower postgraduate exam pass rates (66%) when compared to those from Hindu, Sikh or those who do not follow a religion (70%).
- Make up 6.5% of the general population but 10% of medical professionals.

WHY WE MADE A GUIDE

There's an attainment gap, locally and nationally

We recognised we could do something to help

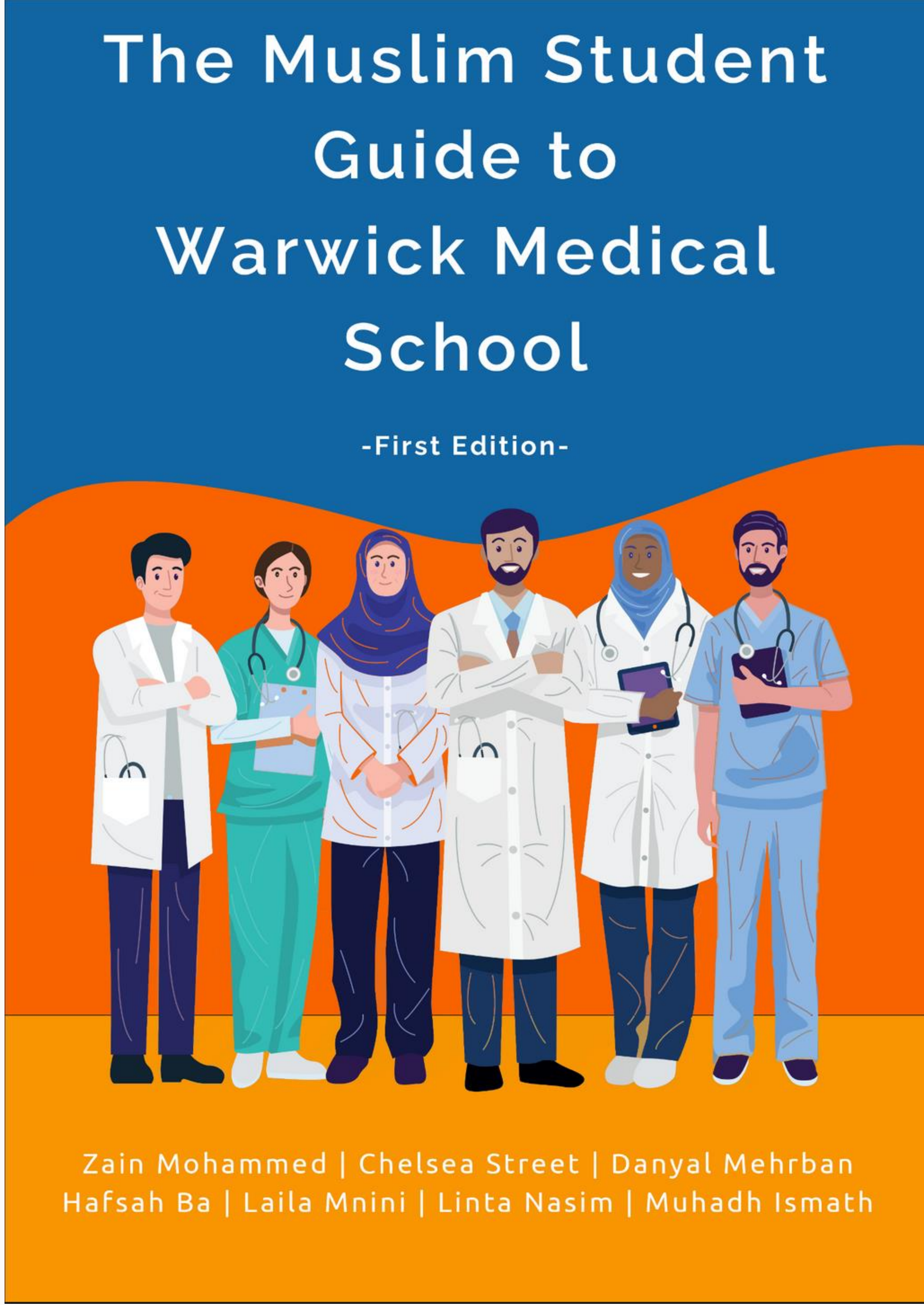

A Year in my Hijab

A CBL case about a Muslim student's experience

N.B. The situations mentioned in this case are based on the true accounts of Medical Students around the UK based research done by Students at WMS

How to do CBL

1

Allocate team roles

- Scribe
- Chair
- Researcher

2

Define terms you are not familiar with

3

Develop lines of enquiry

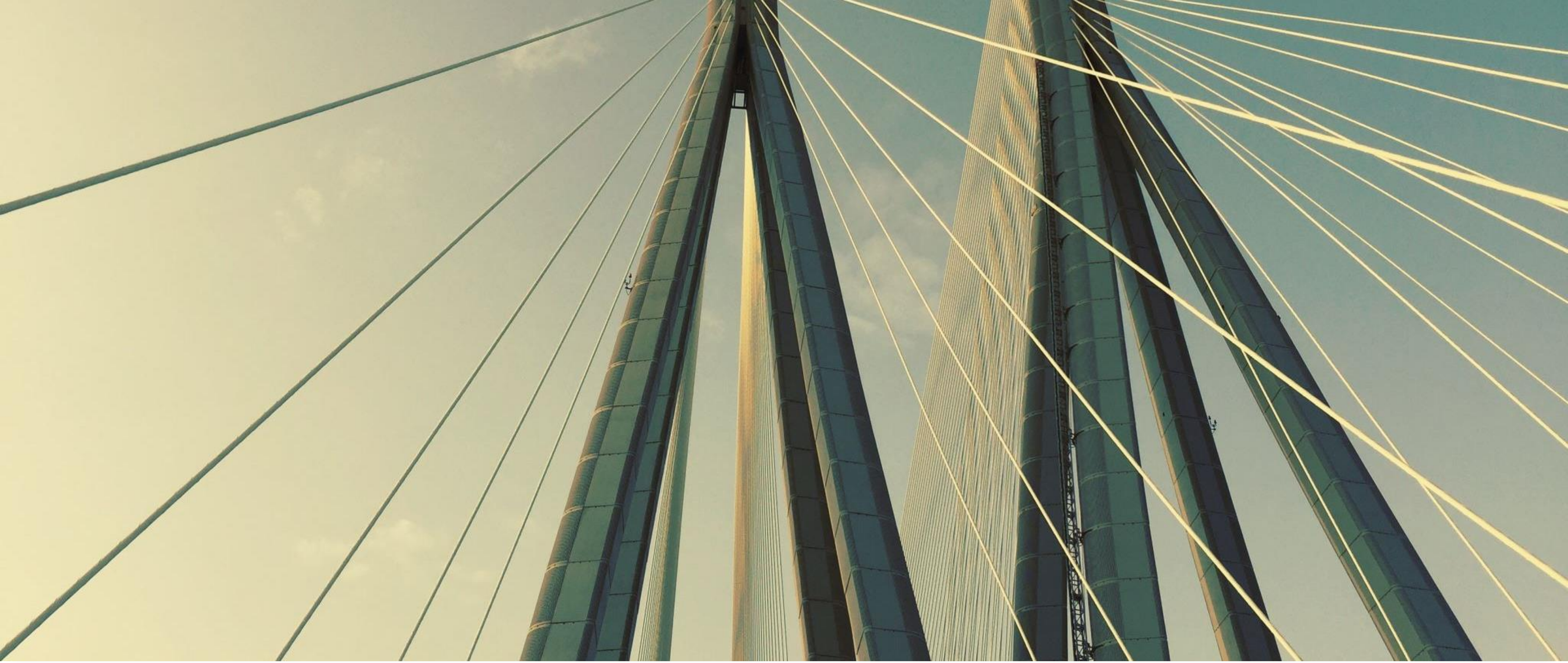

Part 1
Building Bridges: Social Interactions in a Diverse World

Background

Maryam is a Phase 1 medical student excited to join Warwick Medical School in 2023. She did well with her previous degree in biomedical science, both socially and academically, and she loved the diversity her undergraduate course in Manchester provided.

She has always been interested in surgery and keen to learn more about anatomy and clinical skills.

She considers herself a practising *Muslimah* who prays *Salah* regularly, does not drink alcohol, fasts in Ramadan, observes a *halal* diet and wears a *hijab*.

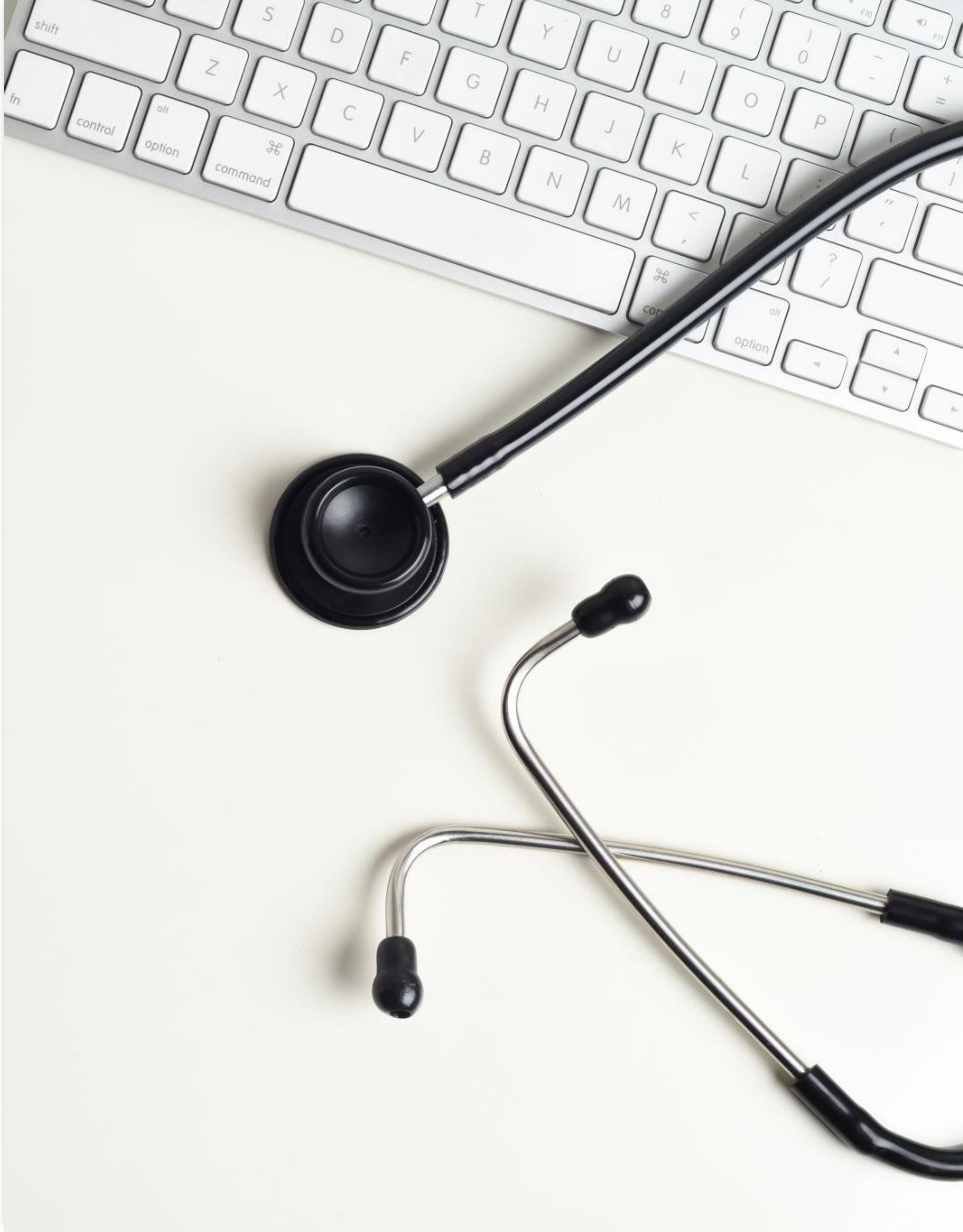

Case-Based Learning

She gets on well with her Case-based learning group. She's very confident, taking the chair role for the first session, and thoroughly enjoyed the process.

Following the session, another CBL member asked who would be attending the fresher's party that evening. She stated that she did not plan to attend as she felt uncomfortable. Instead, she suggested that the group have a coffee walk while exploring the campus. The group was accommodating, and they got to know each other well.

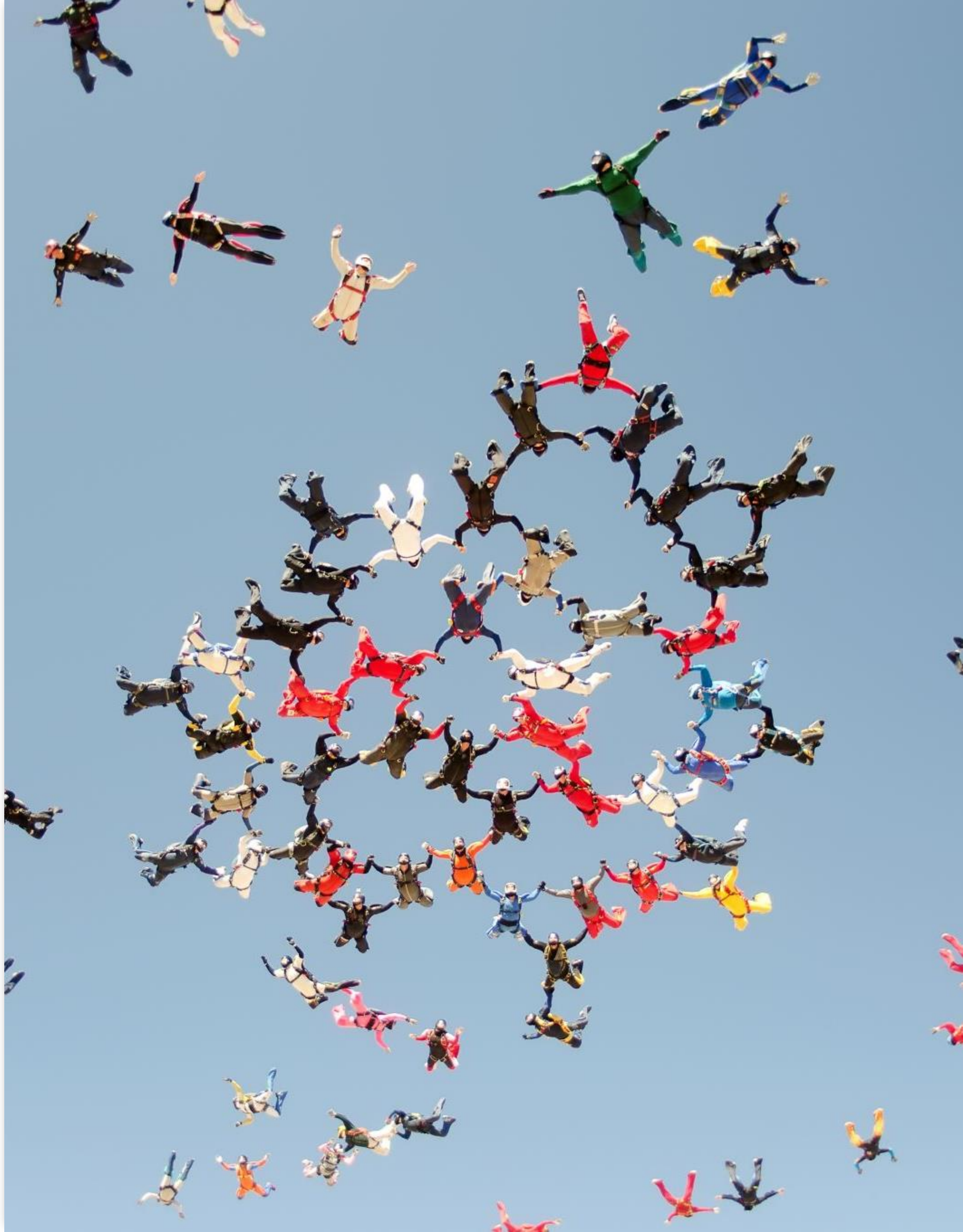

Questions

- Why was this a positive outcome for the group?
- What if she had been more hesitant about her suggestions?
- What would be the implications of her agreeing to attend?
- What would be the best environment for CPT meetings?
- What are some non-alcoholic alternatives for students?
- How can educators facilitate this?

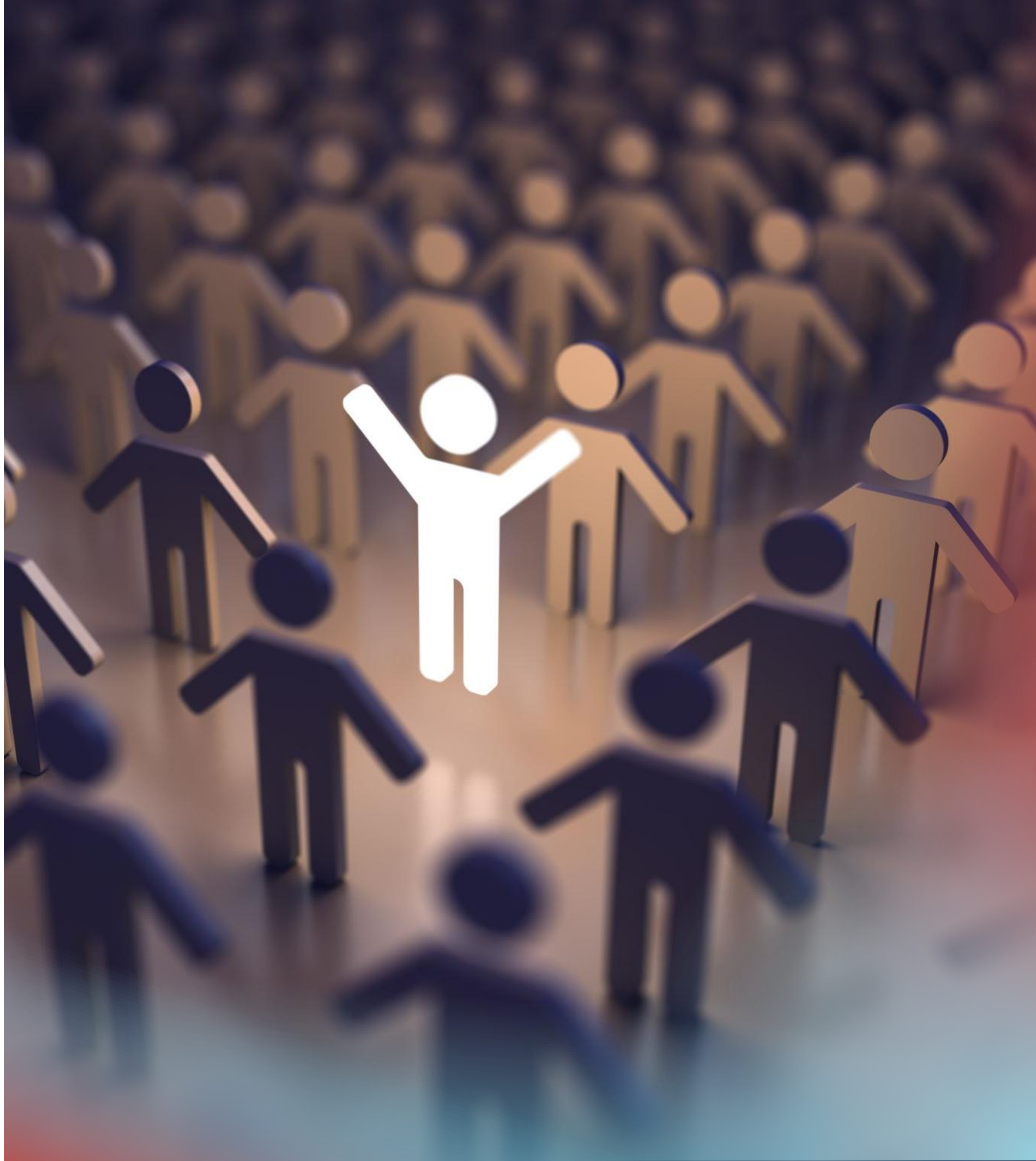

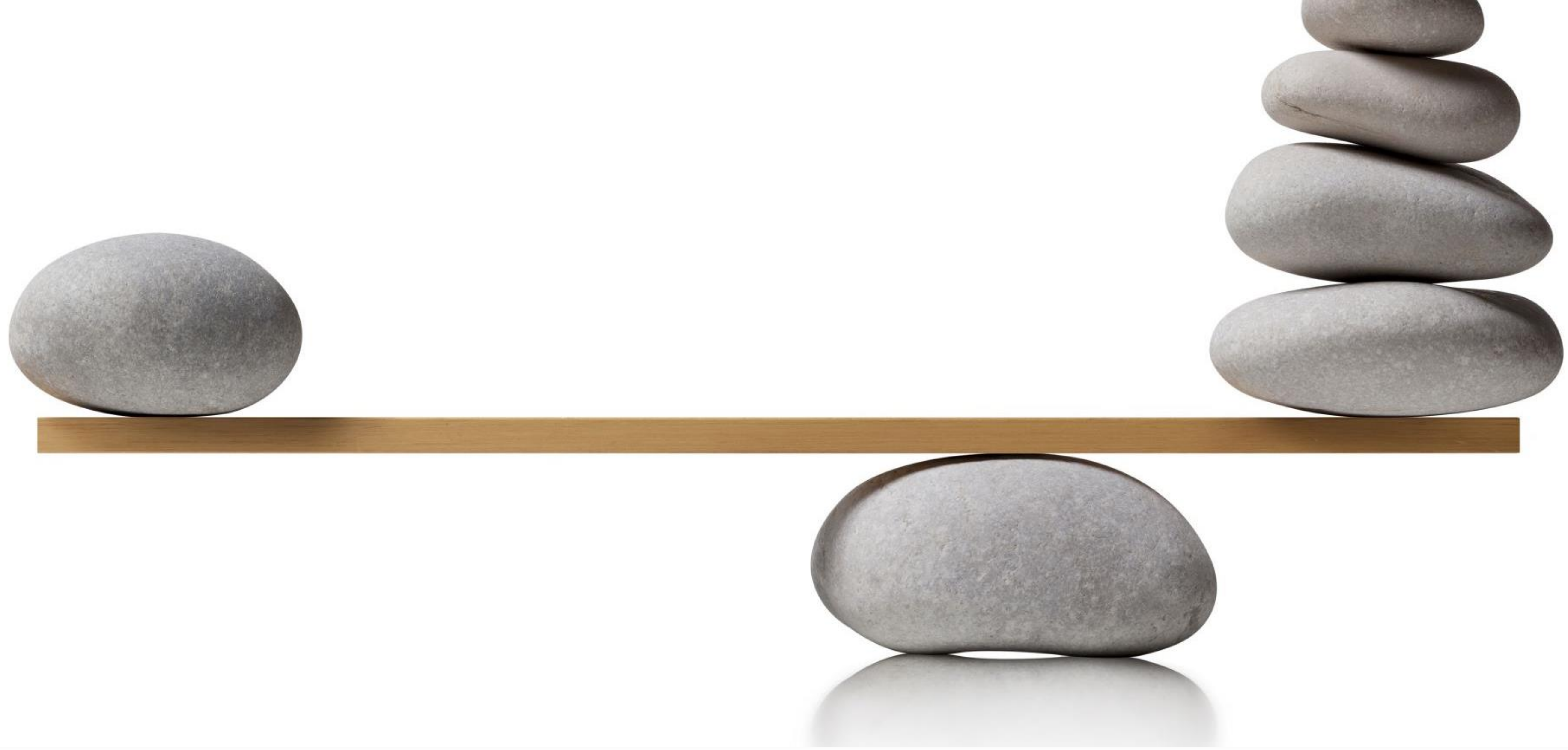

Part 2
Balancing Duties: Integrating Spiritual Practices at school and work

The next day

During her afternoon teaching at the off-site anatomy building, she glances at her watch and shifts uneasily. It is currently 1:55 pm, and she is worried she might **miss** her early afternoon prayer.

She knows that she needs to make *wudhu* before performing her prayers and is worried about where she might perform her ablution and prayer.

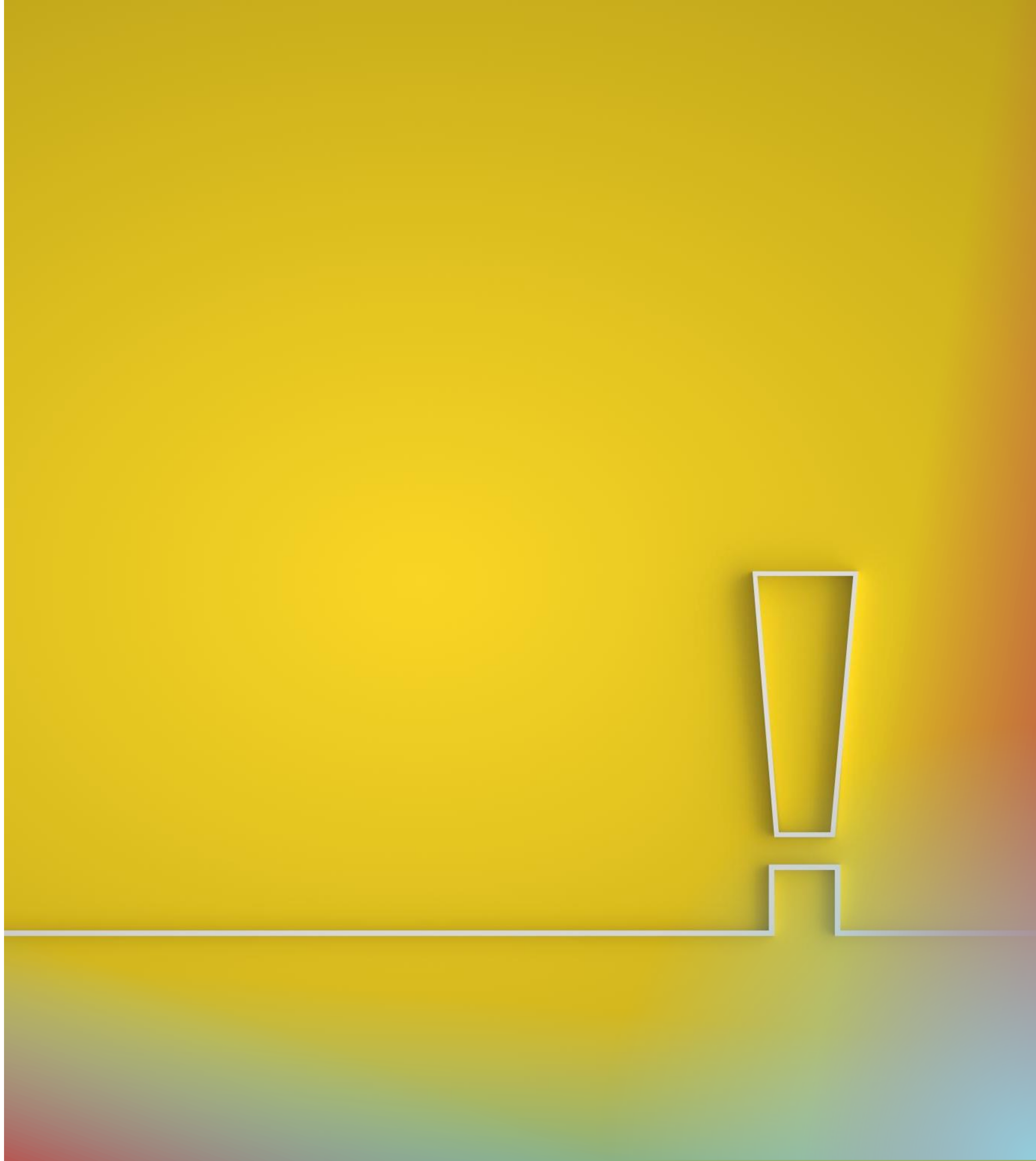

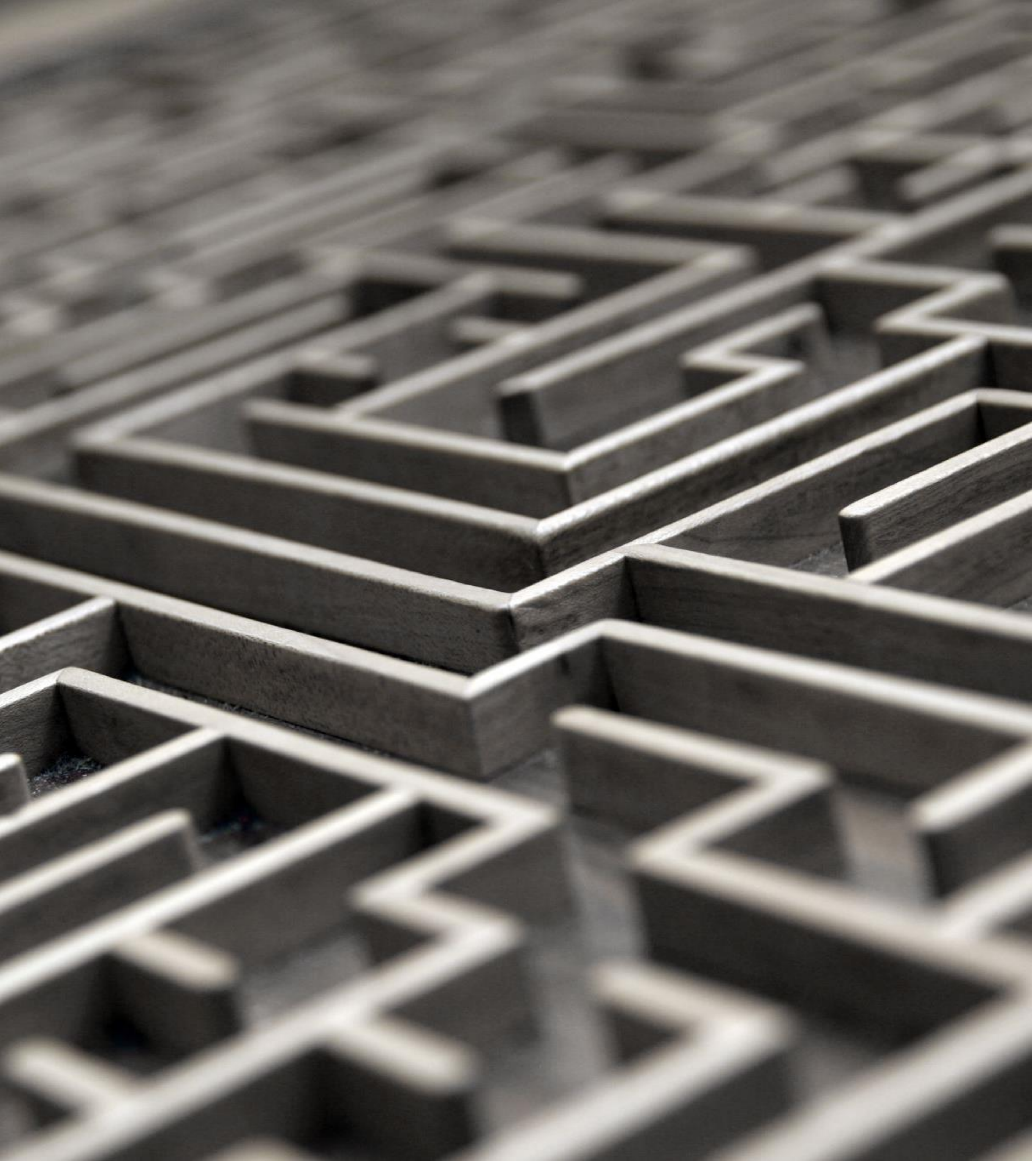

Questions

- Why are Maryam's prayers so important to her?
  - What prayers need to be performed and are they time sensitive?
  - How long do they usually take?
- What challenges might she have with asking to be excused?
- Are there any logistical challenges that she might encounter?
- How is *wudhu* performed?

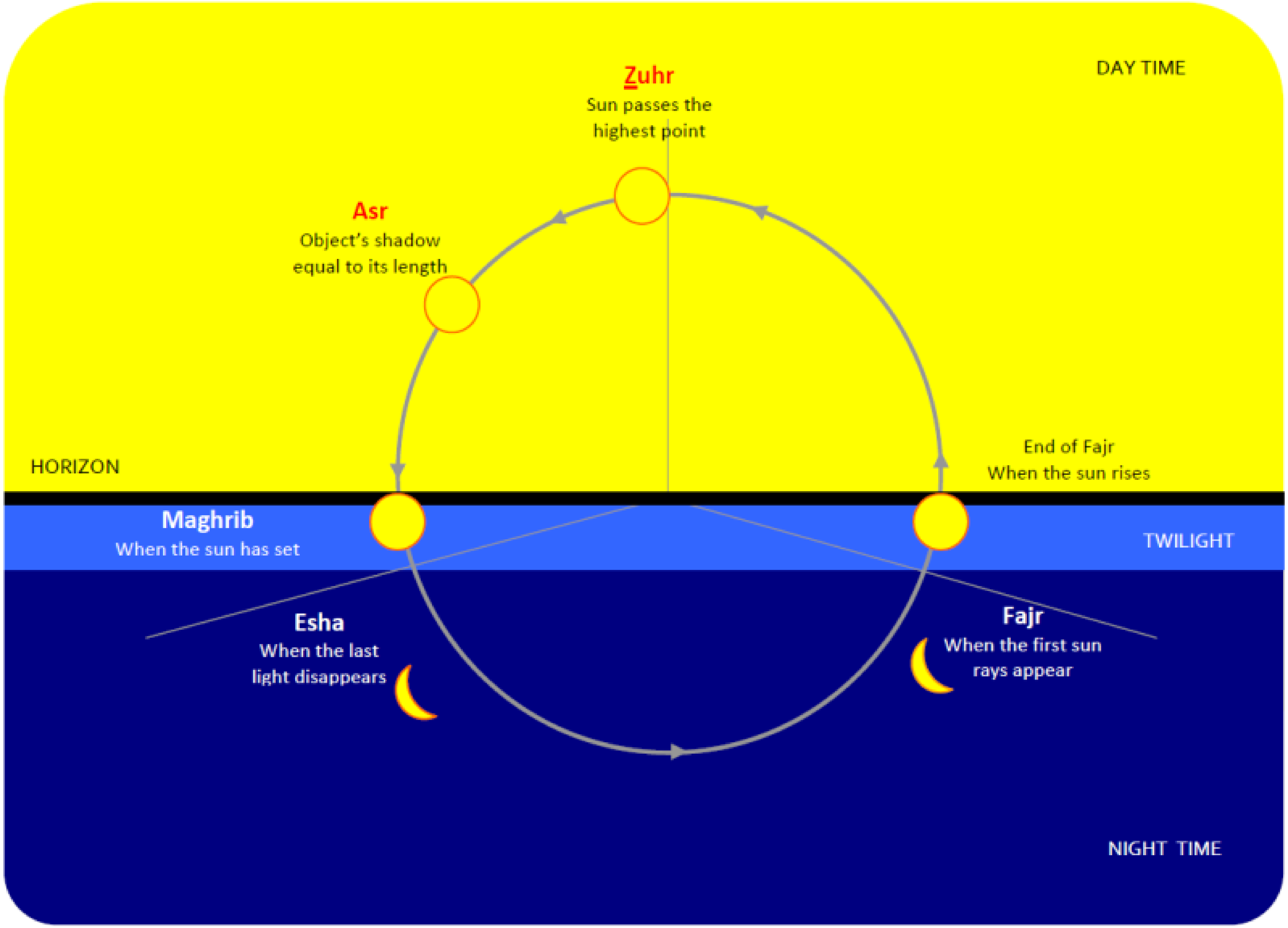

DAY TIME
Zuhr
Sun passes the highest point
Asr
Object's shadow equal to its length
HORIZON
End of Fajr
When the sun rises
Maghrib
When the sun has set
TWILIGHT
Esha
When the last light disappears
Fajr
When the first sun rays appear
NIGHT TIME

Prayer Times December

Prayer name	*Prayer time*
Fajr	06:00
Sunrise	07:42
Dhuhr	11:54
Asr	13:37
Maghrib	15:58
Isha	17:35

Prayer Times August

Prayer name	*Prayer time*
Fajr	03:40
Sunrise	05:35
Dhuhr	13:22
Asr	17:23
Maghrib	21:00
Isha	22:01

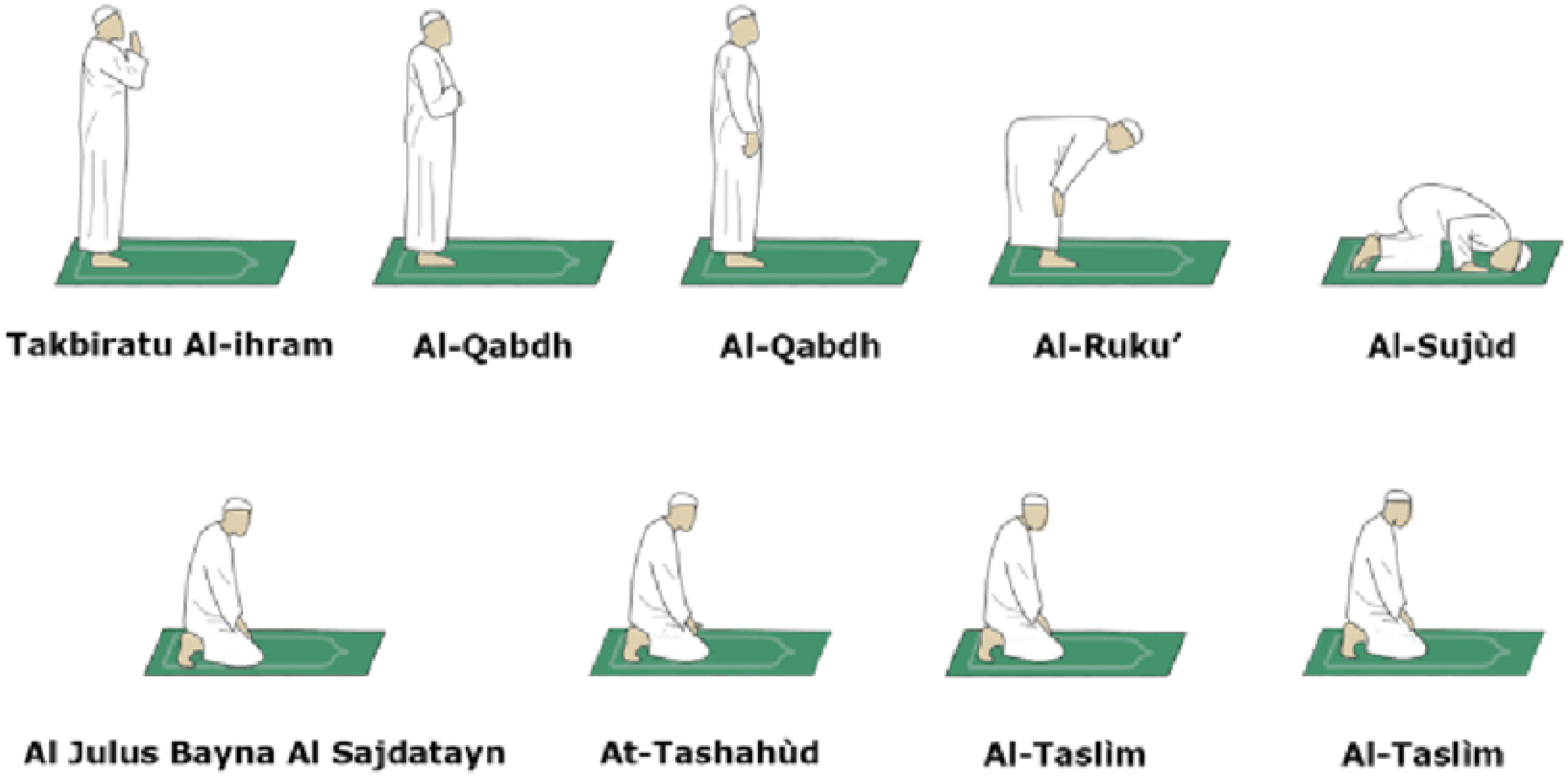
Takbiratu Al-ihram
Al-Qabdh
Al-Qabdh
Al-Ruku'
Al-Sujùd
Al Julus Bayna Al Sajdatayn
At-Tashahùd
Al-Taslìm
Al-Taslìm

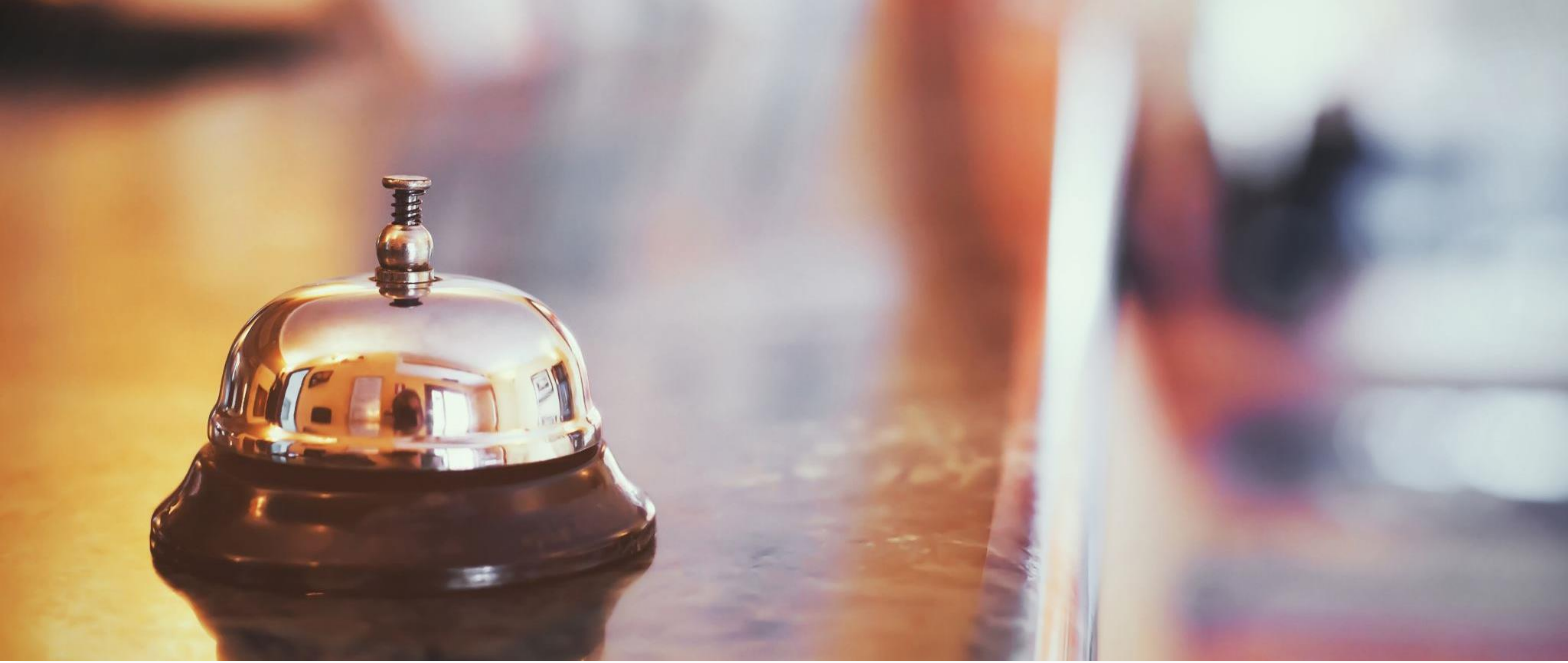

Midway Pause: Recharge and Regroup

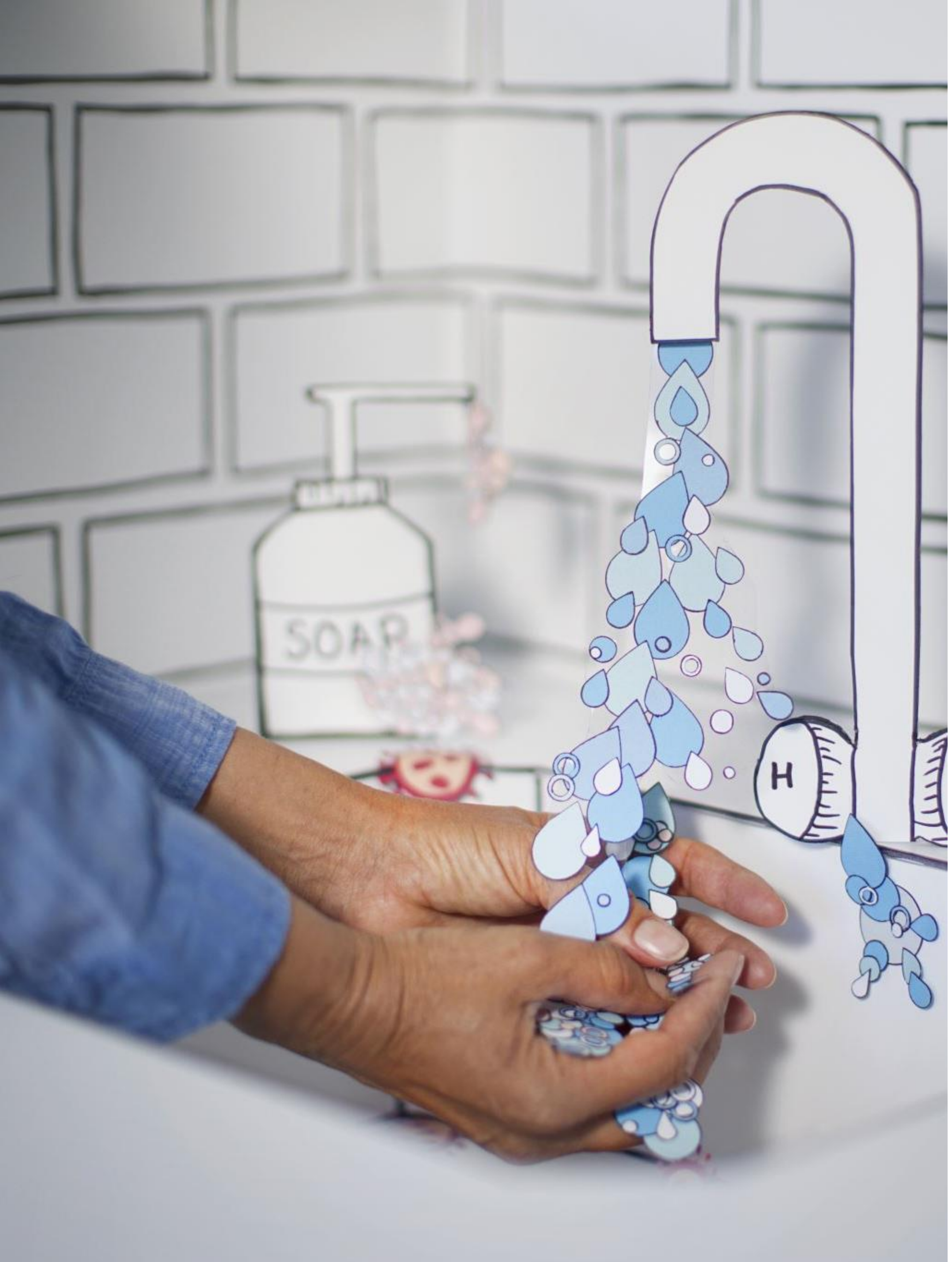

Prayer

She performs her ablution in the nearby showers by washing her **hands**, **face, forearms, and feet thrice and wipes over her hair**, for which she needs to remove her hijab, hence uses a private cubicle.

This suits her as she doesn't want to wash her feet in the sink. On a previous occasion, a colleague walked in and gave her a strange look.

She quickly performs her prayers in the changing room using the portable prayer mat in her bag. She enjoys studying at the Main Medical School campus as she has access to the multi-faith space, where she finds a peaceful and quiet environment to reflect.

Questions

- Are there any groups of students that couldn't use the shower/sink facility to perform ablution?
- How do daily prayers differ from Friday prayers?
- How would Muslim clinicians manage with prayers when at work?

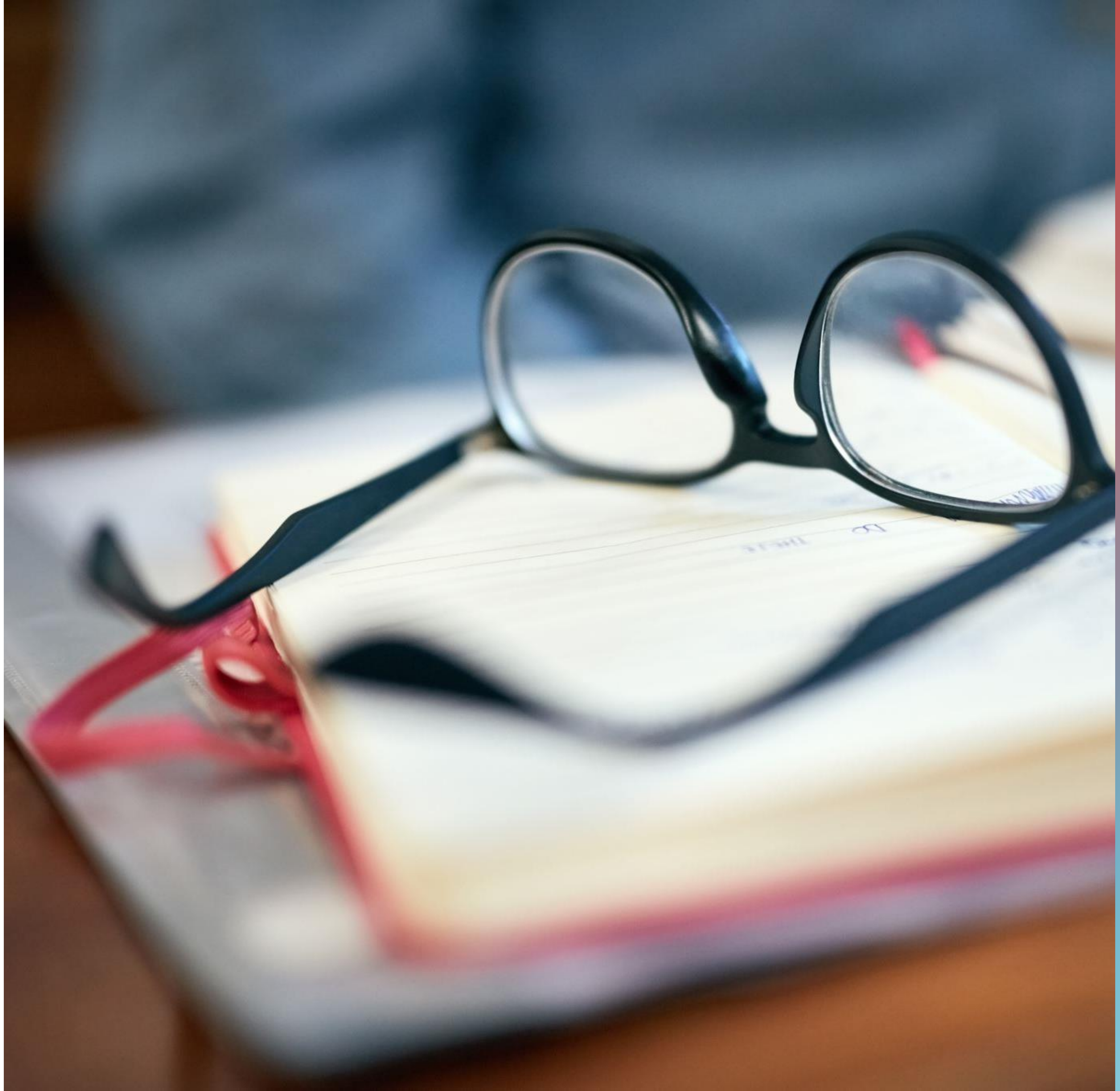

How WMS has supported students with prayers

- Established a new purpose built multi faith space in 2023
  - Original room: Smaller, not enough resources
  - Current room: Larger, enough prayer mats and scarves, barrier provided
- Signs for ablution
- Has invited students to reorganise sessions to facilitate their prayers especially Fridays.

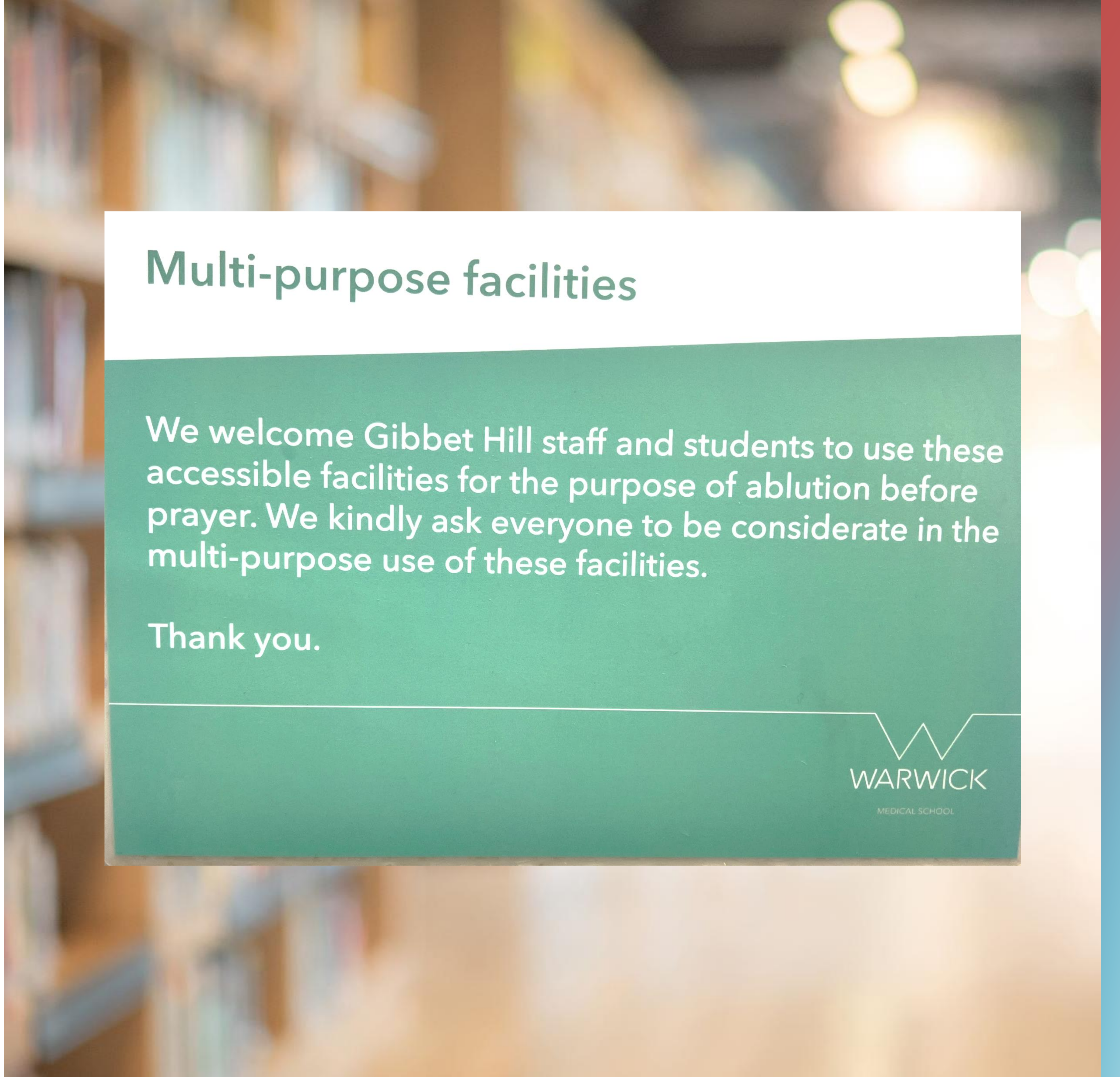

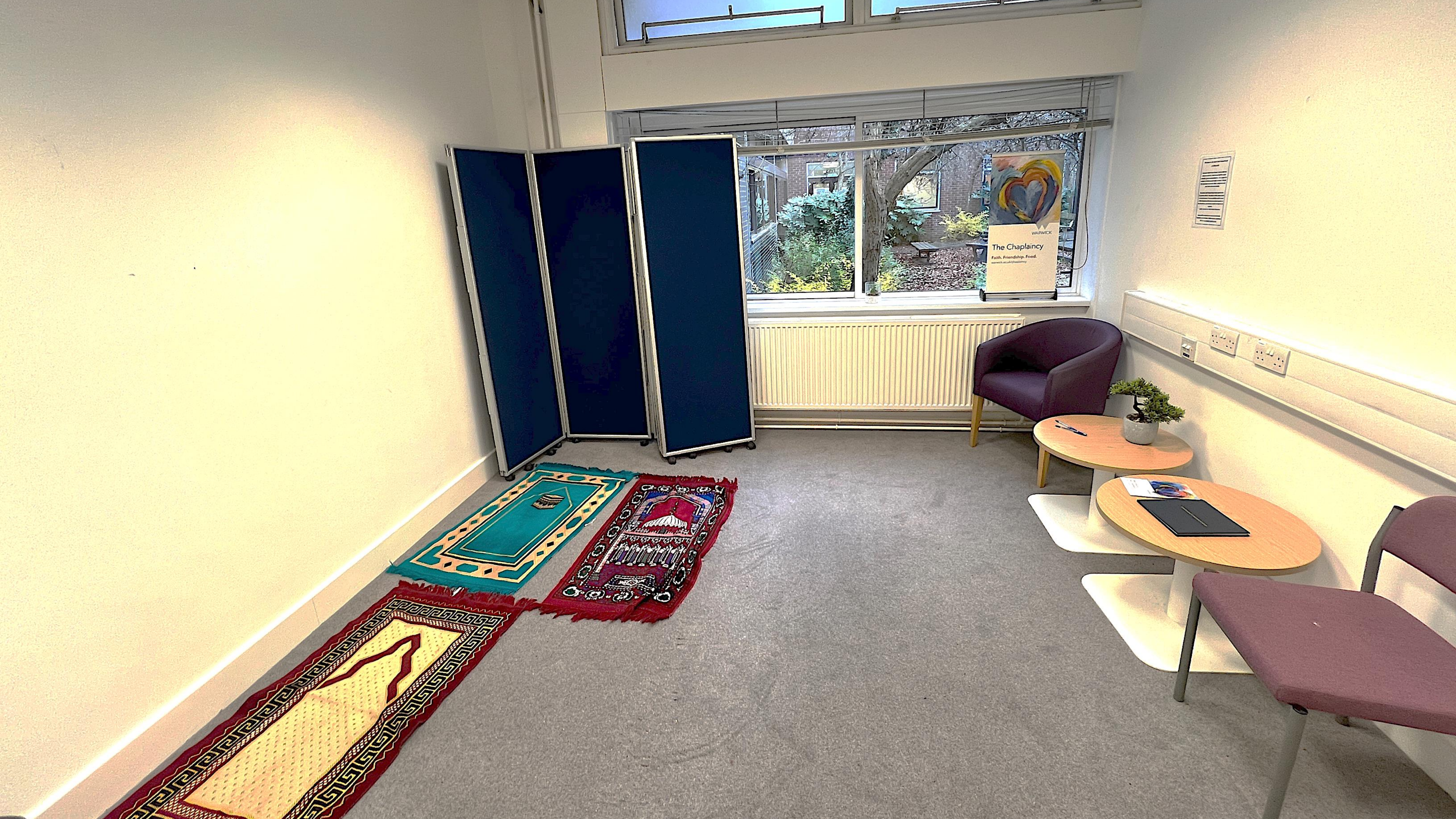
WARWICK
The Chaplaincy
Faith. Friendship. Food.

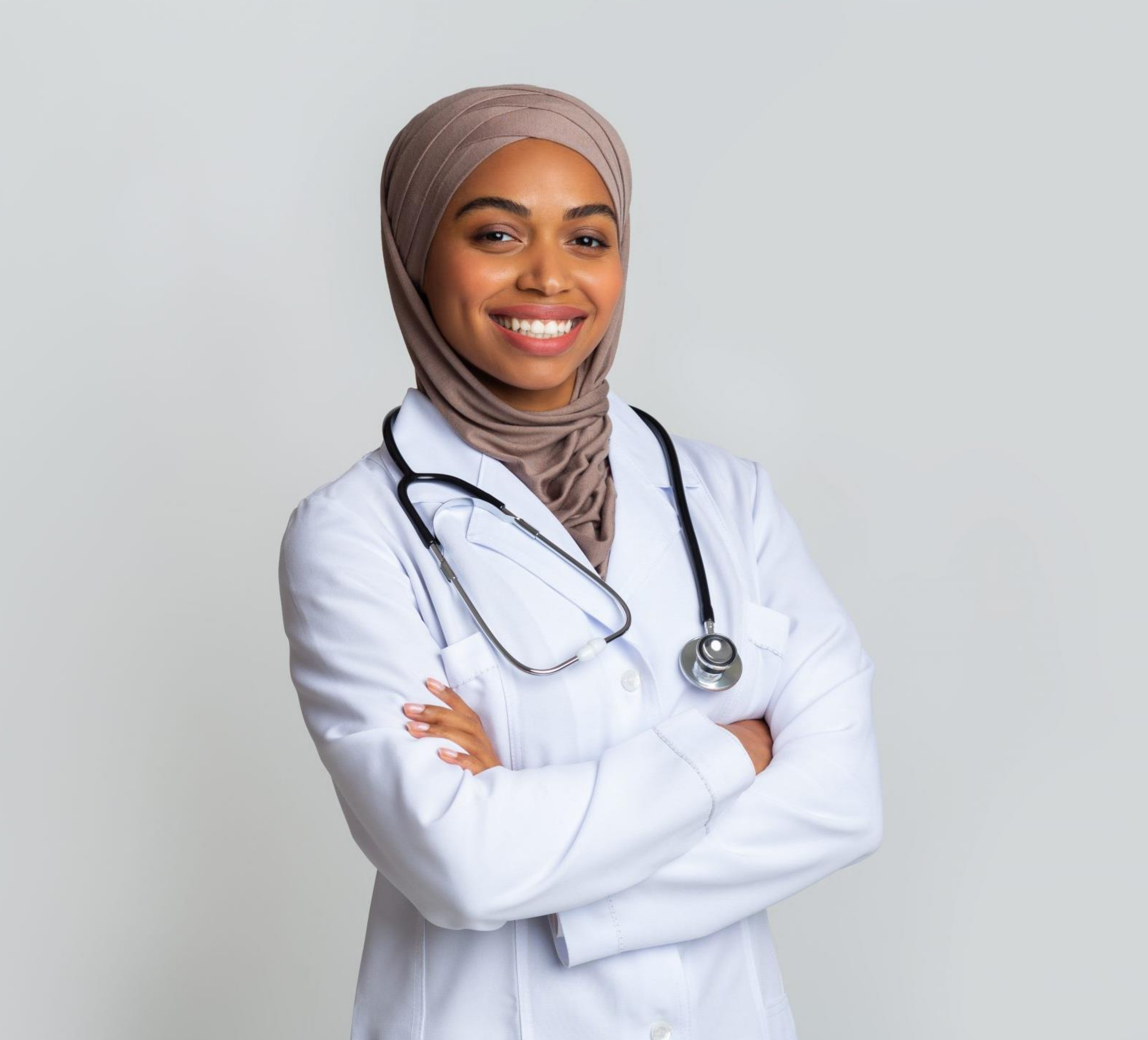

Part 3

The Hijab and Professional Identity: Finding Common Ground

or

Integrating Hijab: Challenges and Solutions in Professional Settings

Clinical Skills

The group is learning how to do a hip examination during clinical skills. There is a simulated patient present for the demonstration who is wearing shorts. She starts to worry as the facilitator mentions the importance of correct exposure for the exam.

She doesn't want to expose herself in front of everyone as she is observing *hijab* but worries about her colleague missing out on the opportunity to examine her correctly.

Looking around, she notices curtains that can be pulled around each couch and is relieved.

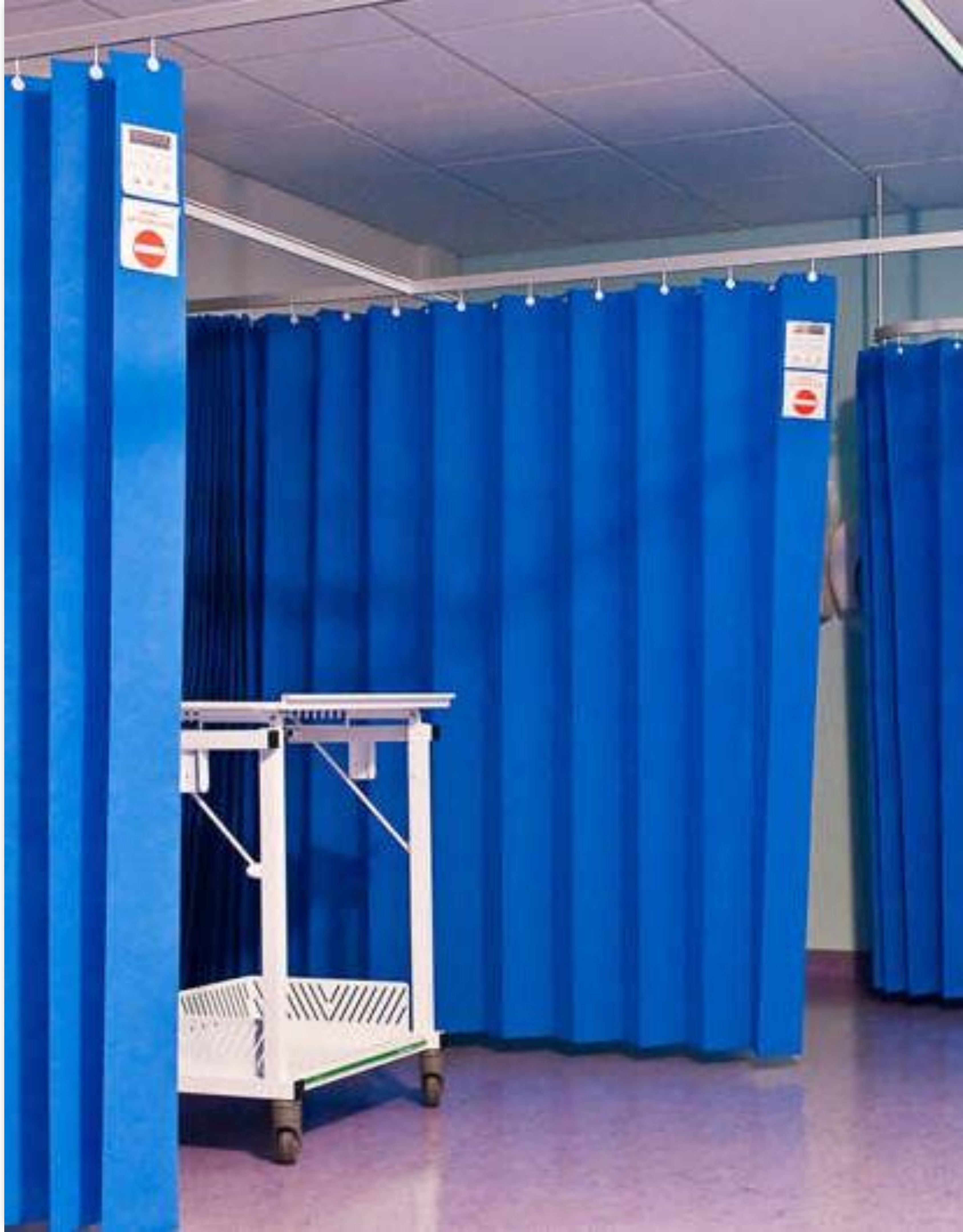

Questions

- **Why would Maryam not want to expose herself for the examination/demonstration?**
- Why would she not feel comfortable volunteering for a manual BP demonstration?
- **How might the power dynamics between students and staff affect the authenticity of the consent they provide when being asked to volunteer for clinical skills?**

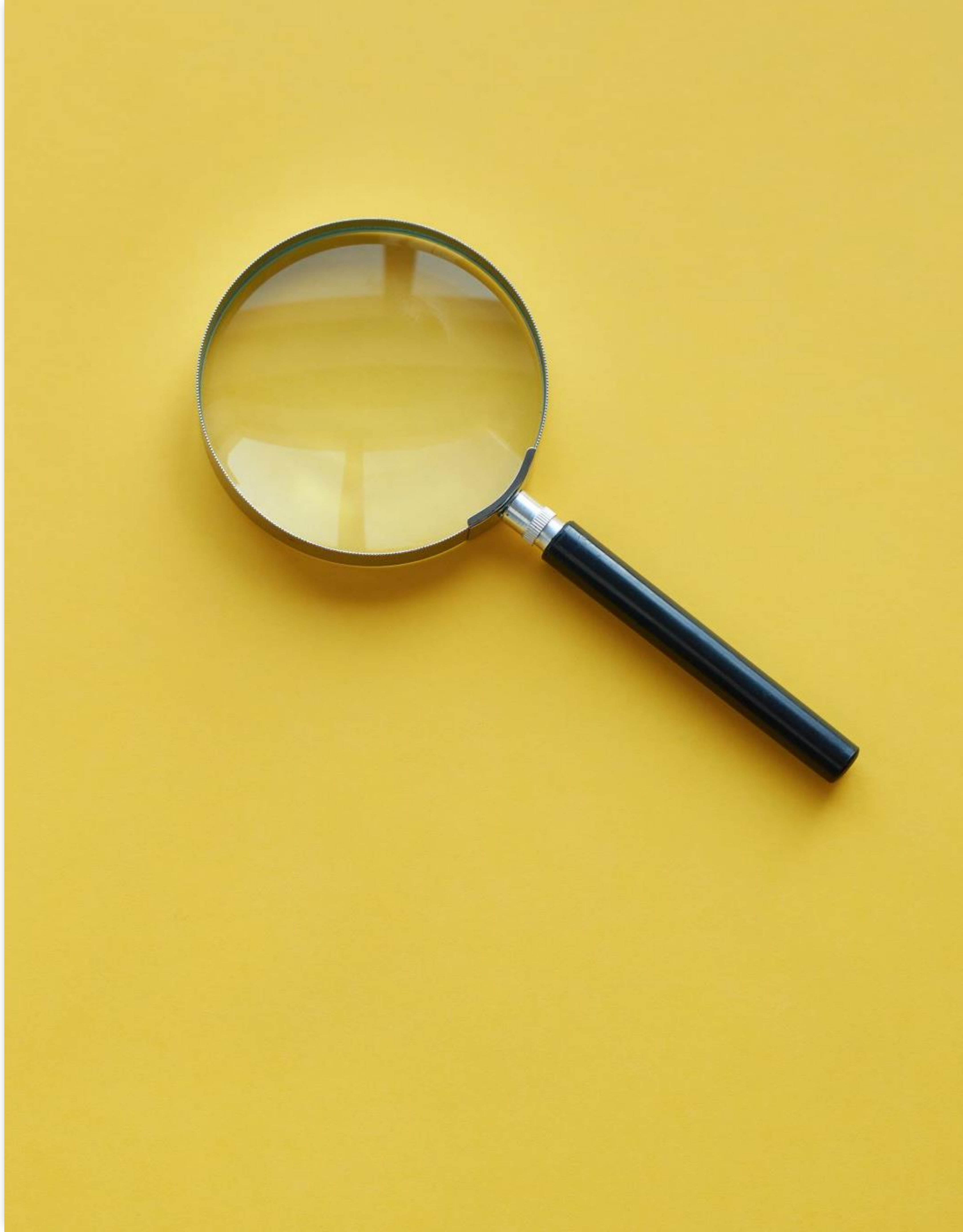

Theatre

She attends scrubbing training and is excited to join her first case. She doesn't remember whether the training mentioned her hijab but placed a theatre cap over her headscarf.

Moving towards the sterile field, someone shouts, 'You can't have that on in here!'. She goes slightly blank, apologises, and steps away from the field. She hadn't thought her *hijab* would be a barrier in her training.

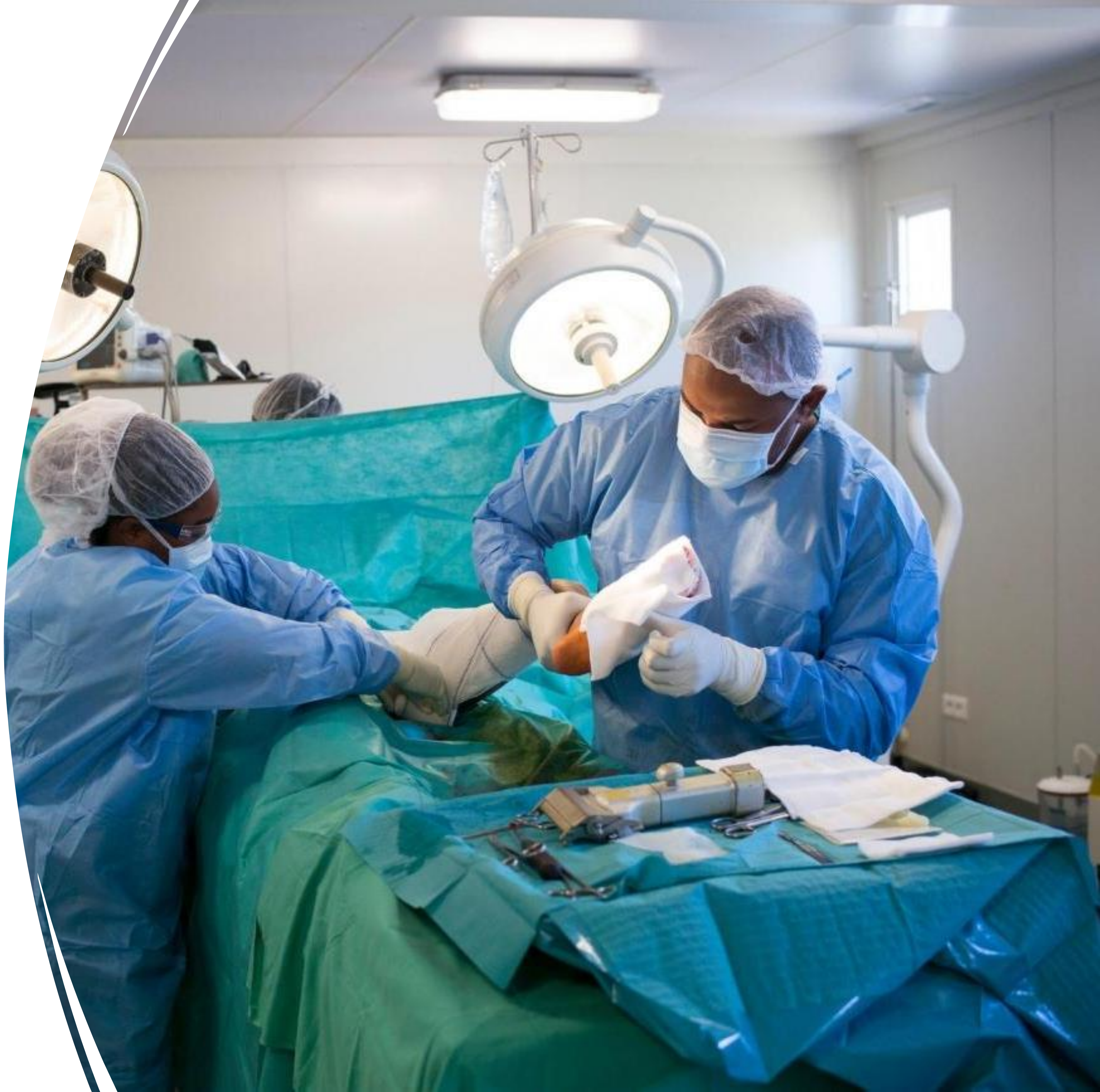

Questions

- **What is the function of a scrub cap?**
- **Can hijabs be worn in theatre?**
- What is the ruling on bare-below the elbow in the clinical environment?
- How should Maryam have handled the situation?

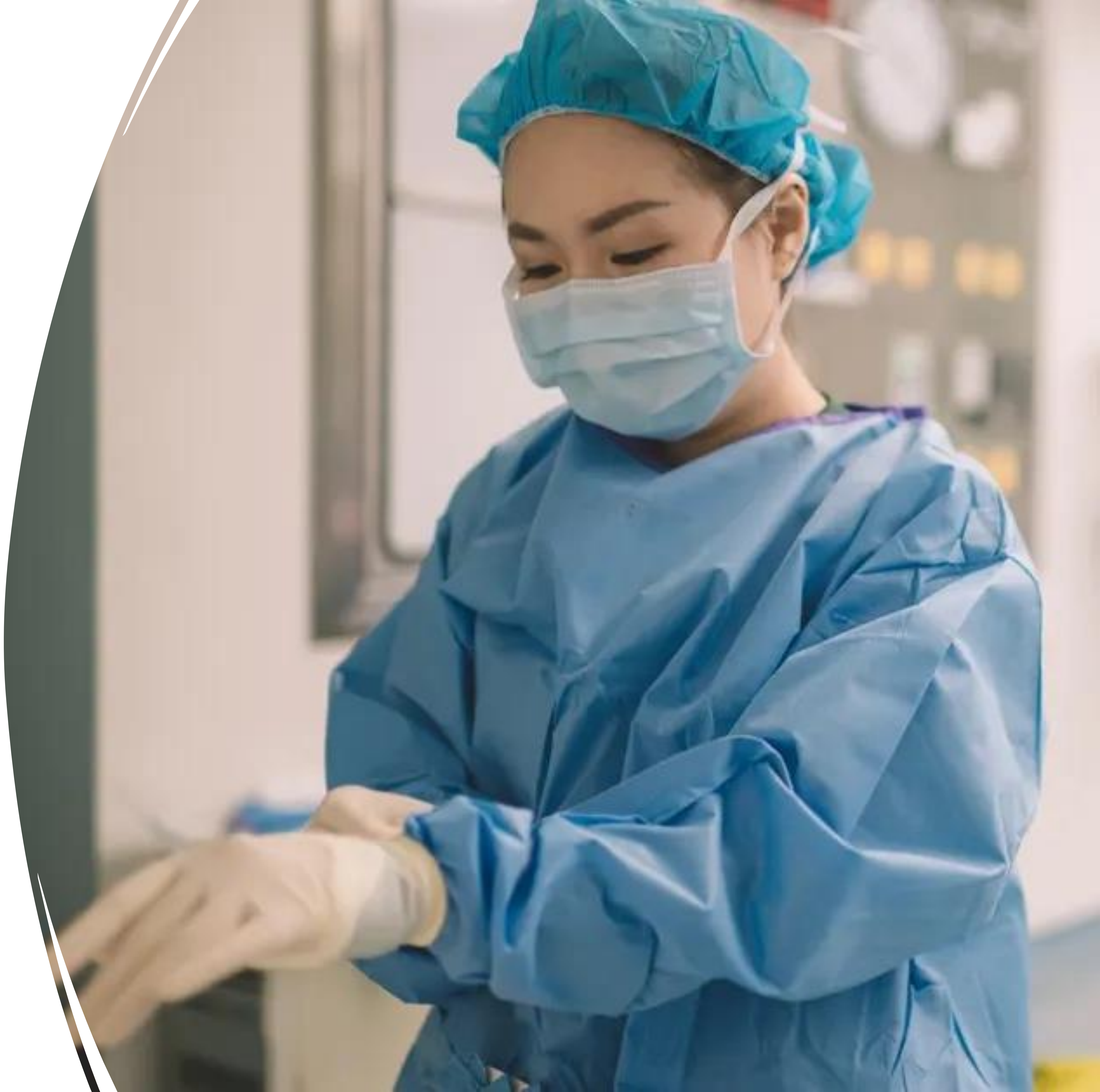

Theatre

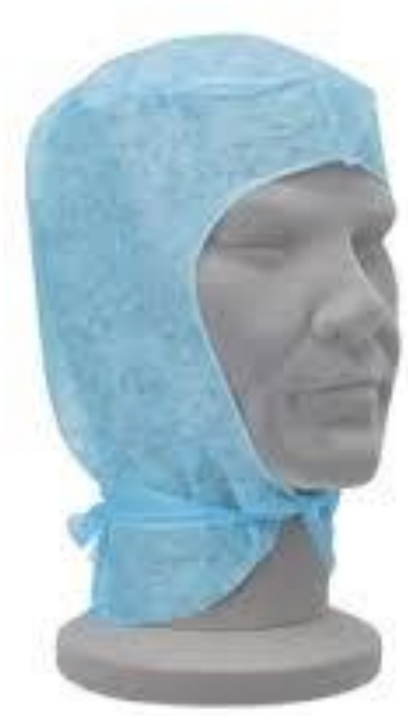

- She later was made aware by a senior medical student that whilst disposable theatre *hijabs* are available in some trusts, they are not widely available.
- The best option is to keep a separate clean hijab washed at 60 degrees specially for theatres.
- An alternative would be a sterile hood that is often used in orthopaedic theatres, which she is excited to use.

Royal Derby Hospital: Disposable sterile hijabs introduced

17 December 2019

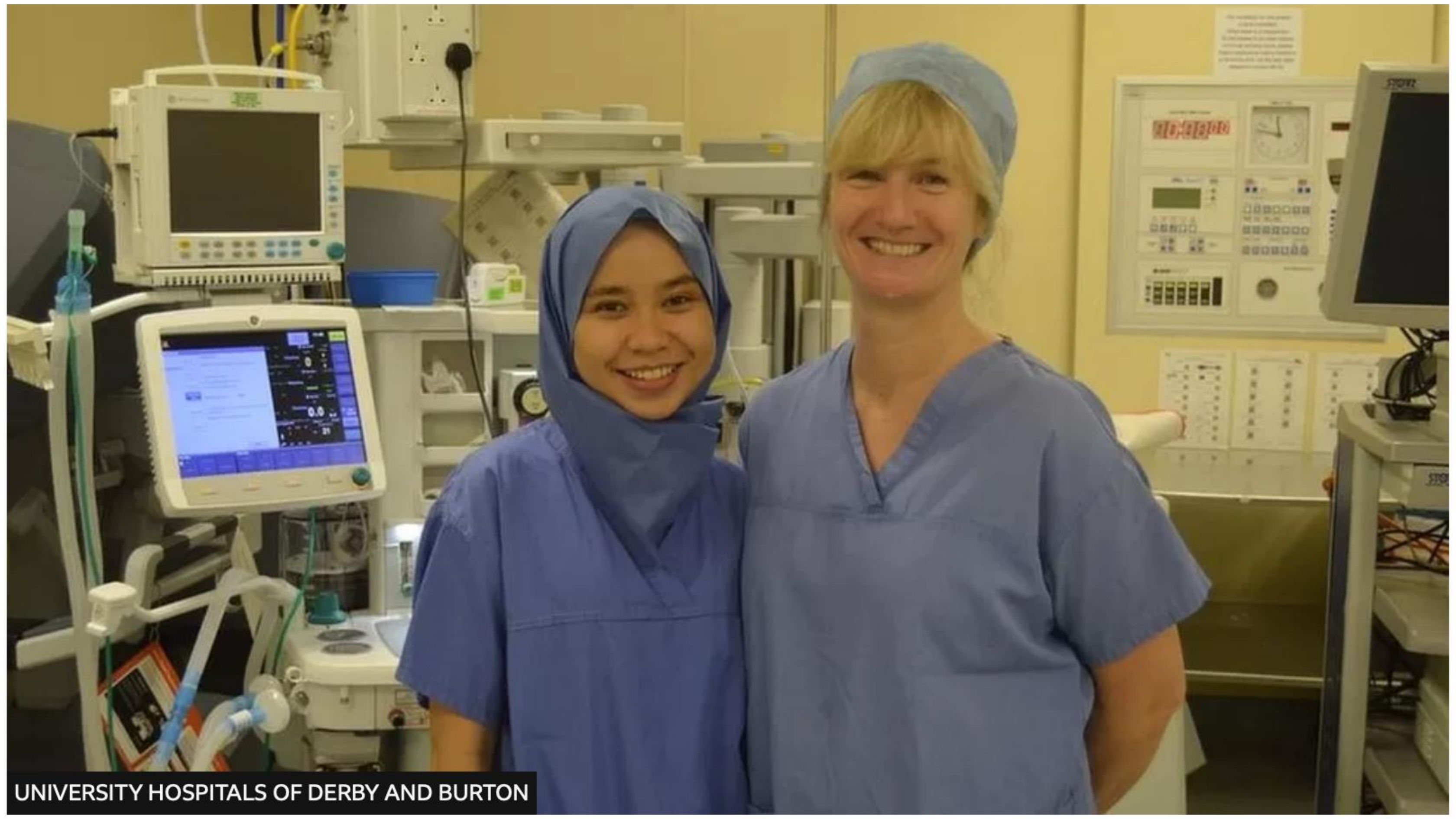

Junior doctor Farah Roslan (left) with consultant surgeon Gill Tierney

A hospital trust believes it is the first in the UK to introduce disposable sterile headscarves for staff to use in operating theatres.

Junior doctor Farah Roslan, who is Muslim, had the idea during her training at the Royal Derby Hospital.

She said it came following infection concerns related to her hijab that she had been wearing throughout the day.

It is hoped the items can be introduced nationally but NHS England said it would be up to individual trusts.

In Clinic

She attends a clinic with a consultant and arrives early. Her clinic partner arrived 10 minutes late into the clinic.

Strangely, she did not get much eye contact from the clinician nor many questions directed at her. She began taking her history and mistakenly repeated a question later in the consultation. The clinician interrupted her, stating that it seemed she wasn’t even paying attention. The patient and fellow student were surprised by this- with the patient explaining that it was okay. Her colleague’s history was met with a well done despite forgetting to ask about allergies and family history.

This whole experience was very off-putting for her and left her perplexed.

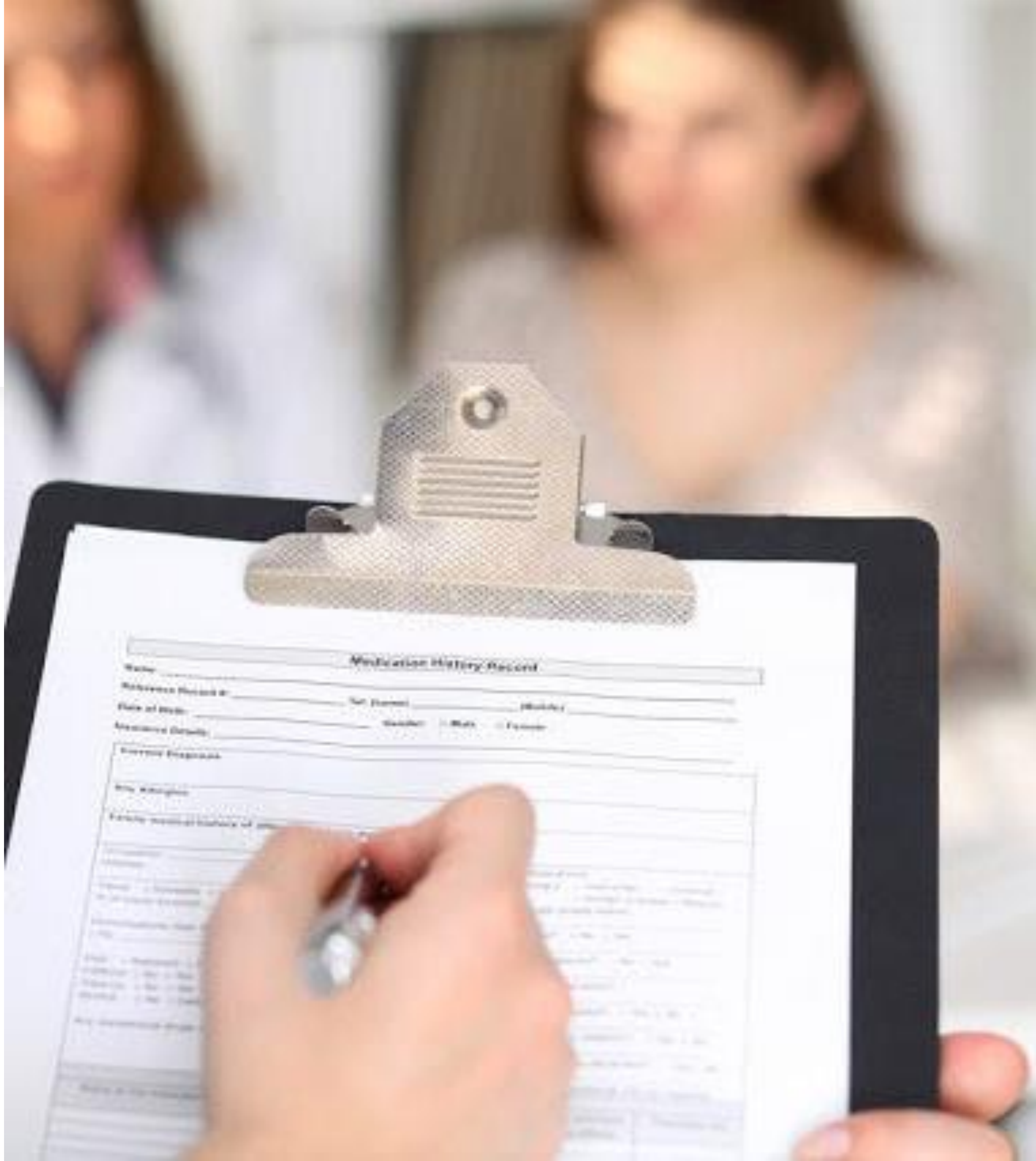

Questions

- How would this make Maryam feel?
- **Who could Maryam turn to for support?**
- **How can she support other students who might be in a similar situation?**

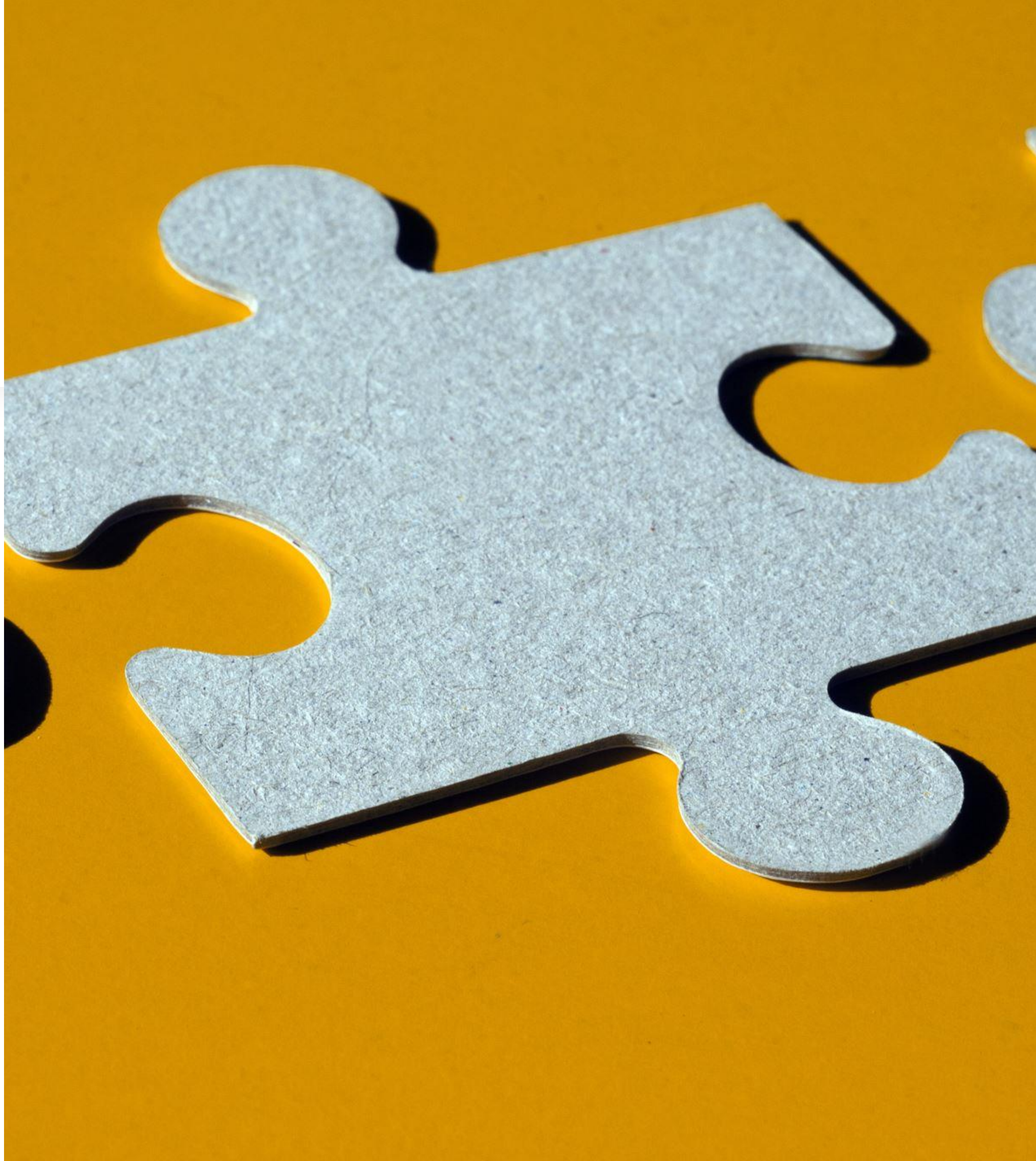

How WMS has supported students

- Established a new purpose built multi faith space in 2023
- Signs for ablution
- Has invited students to reorganise sessions to facilitate their prayers especially Fridays.
- Inclusive CBL cases
- Halal food on campus
- Islamophobia and discrimination pathway
- Clinical skills curtains
- Supported students to develop a Muslim Student’s guide
- SSC2: Multi-centre focus group study exploring the experiences of Muslim medical students.

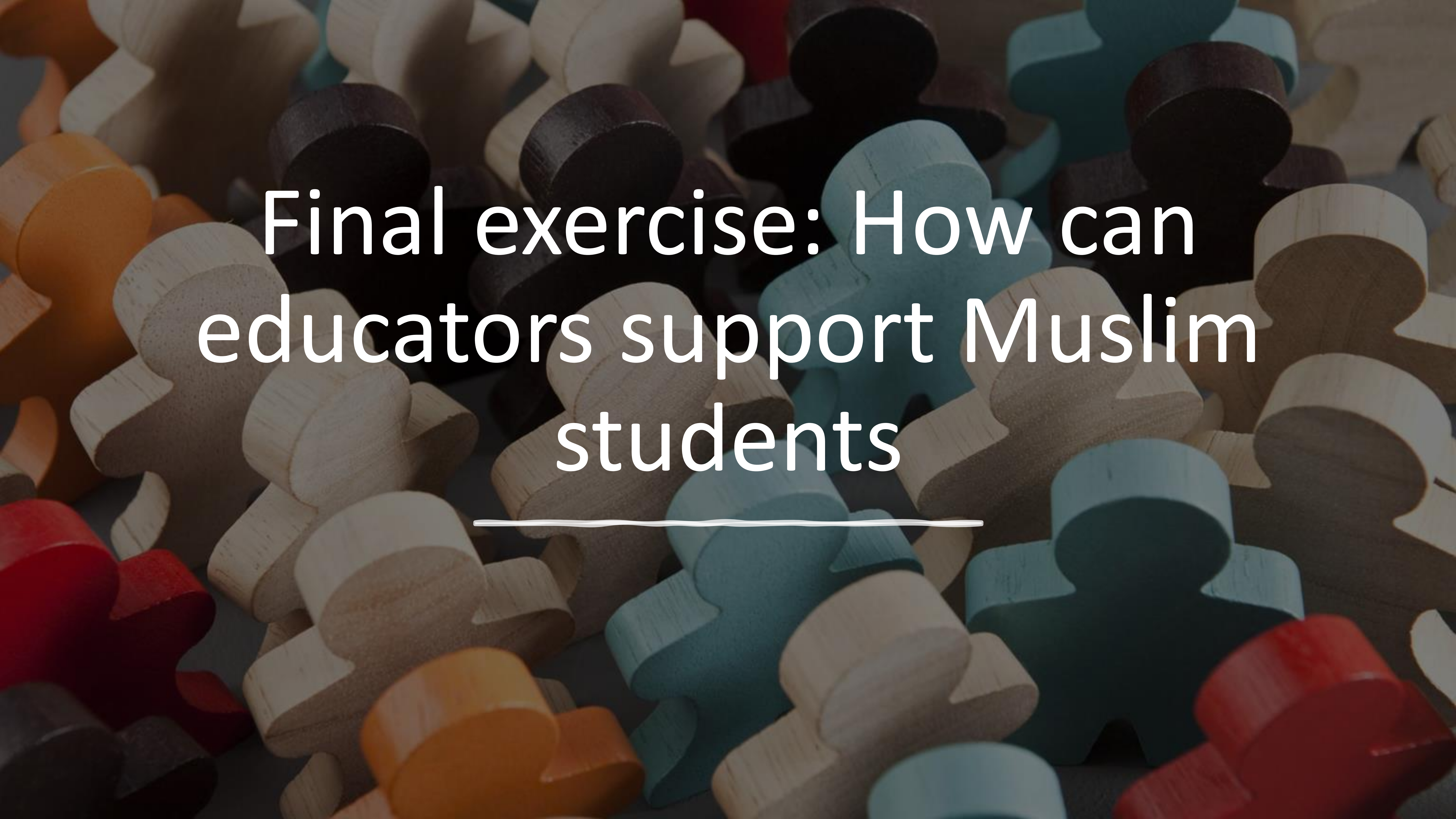

Final exercise: How can educators support Muslim students

Thank you for listening, please leave us some feedback!

Curriculum inclusion

- 150 AD
- 1423 onwards

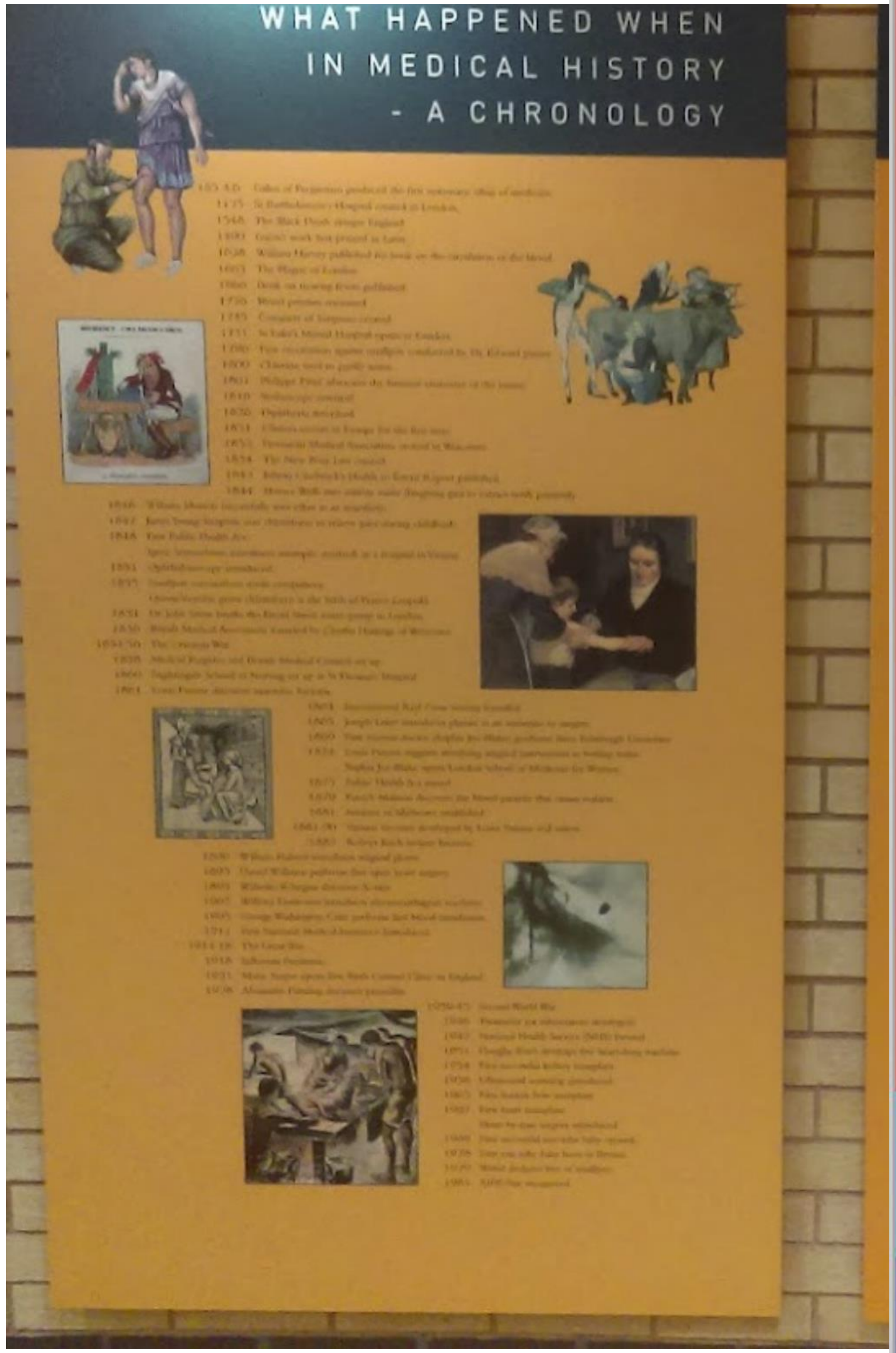

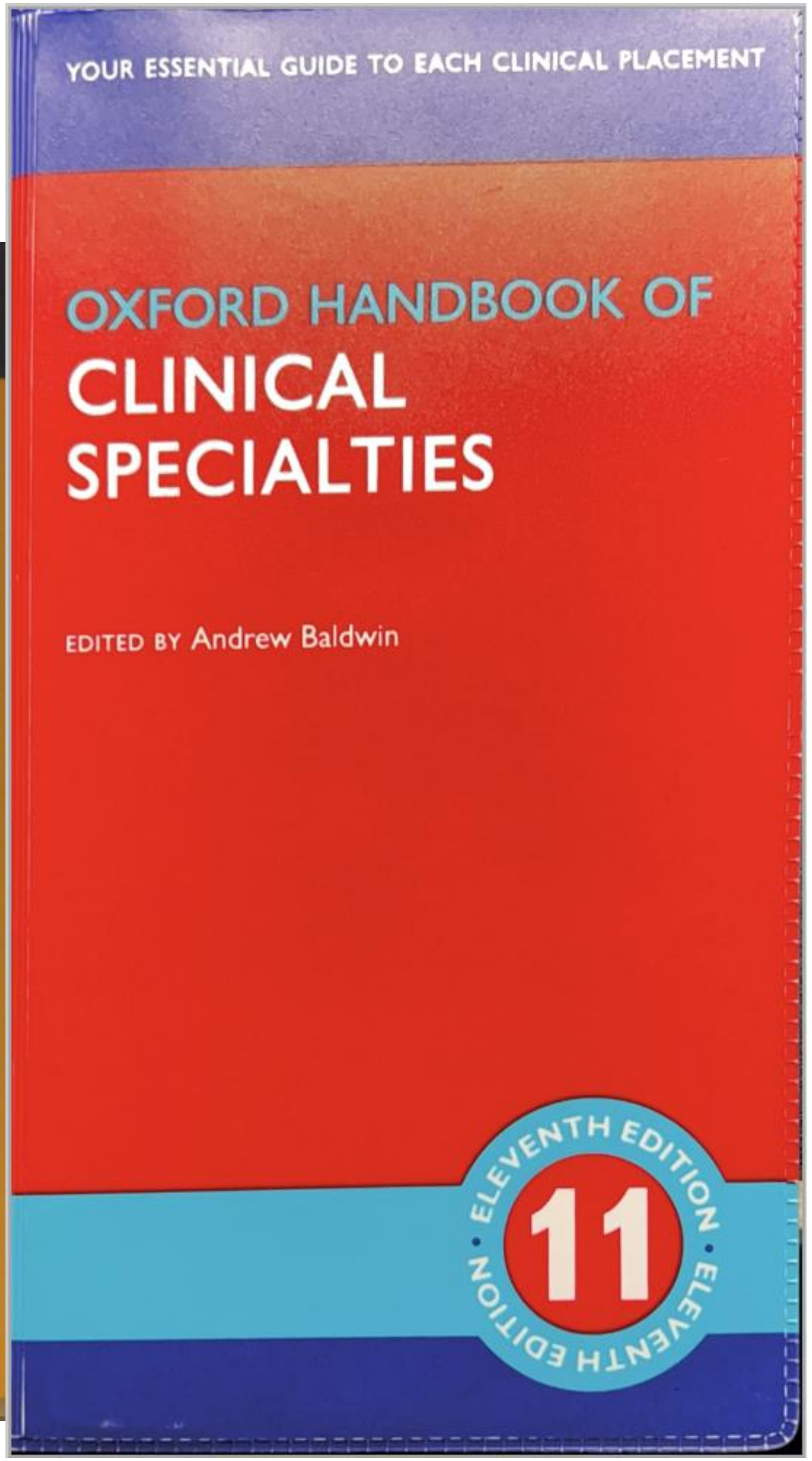

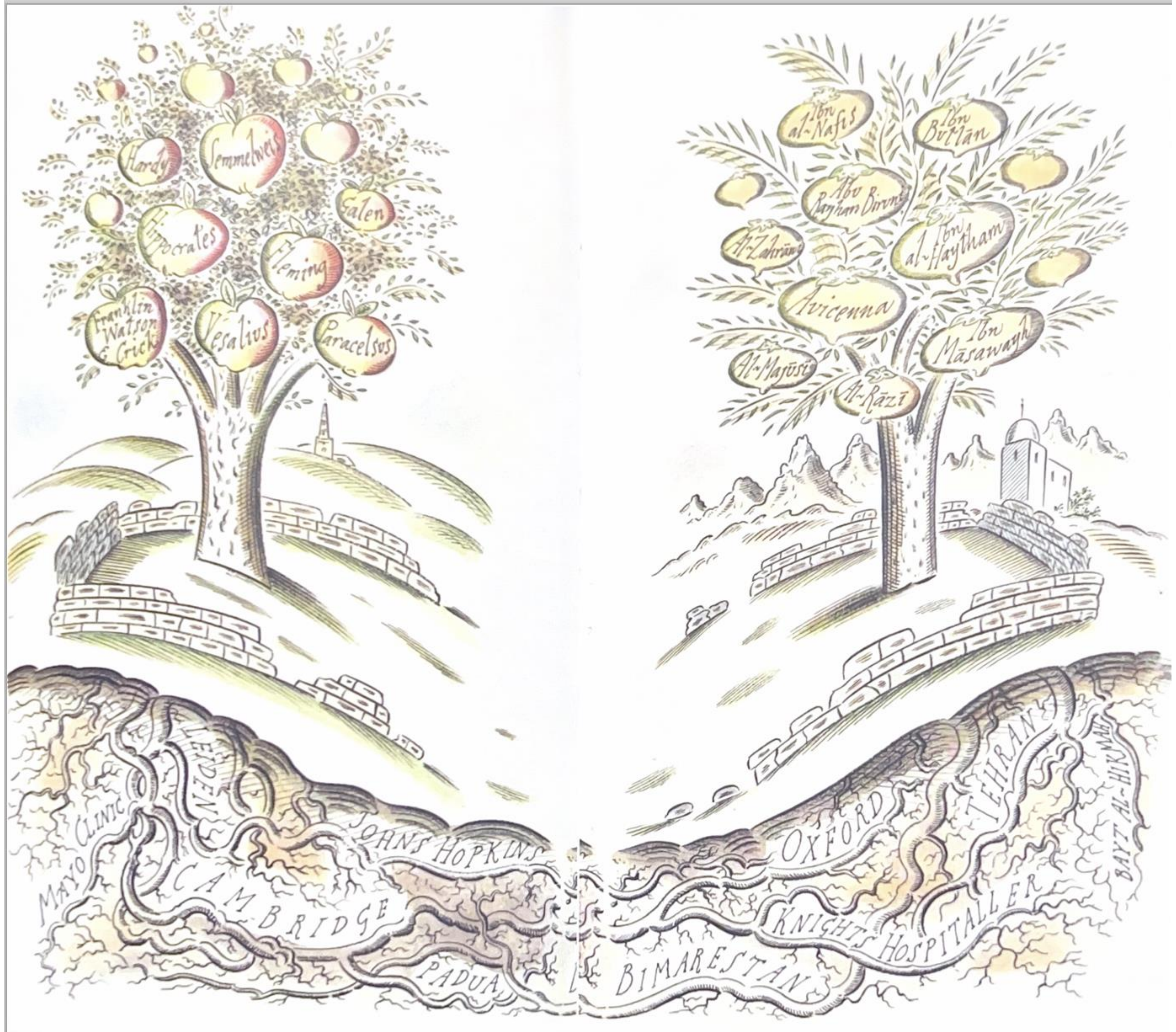

Hardy
Semmelweis
Hippocrates
Galen
Fleming
Franklin Watson & Crick
Vesalius
Paracelsus
Ibn al-Nafīs
Ibn Buṭlān
Abu Rayhan Biruni
Al-Zahrāwī
Ibn al-Haytham
Avicenna
Ibn Māsawayh
Al-Majūsī
Al-Rāzī
MAYO CLINIC
LEIDEN
JOHNS HOPKINS
CAMBRIDGE
PADUA
BIMARESTAN
OXFORD
KNIGHTS HOSPITALLER
TEHRAN
BAYT AL-HIKMAH

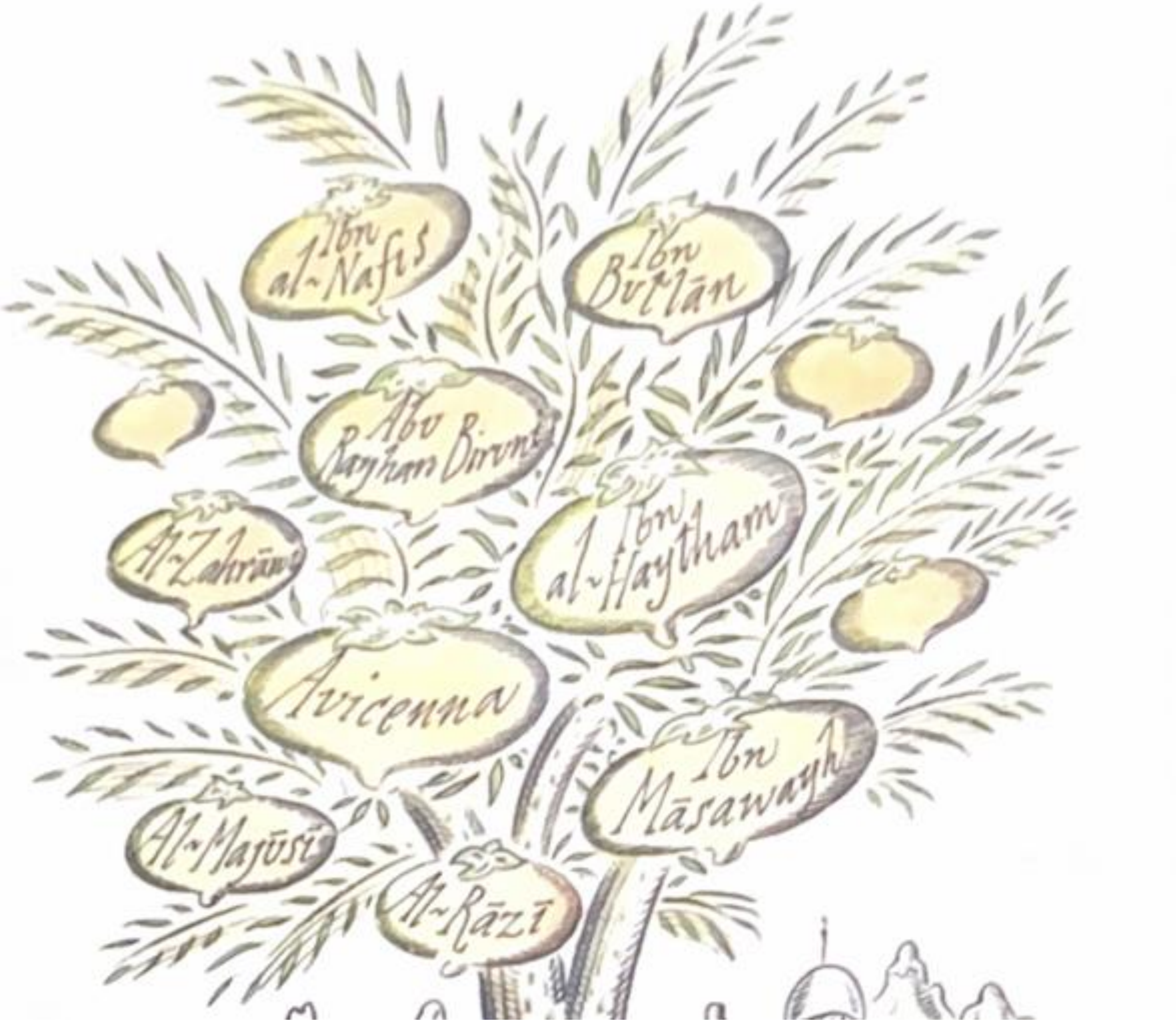

Ibn al-Nafis
Ibn Buṭlān
Abu Rayhan Biruni
Al-Zahrāwī
Ibn al-Haytham
Avicenna
Ibn Māsawayh
Al-Majūsī
Al-Rāzī

Ibn an Nafis

- First described pulmonary circulation in 1200s as:
- *"Blood from the right chamber of the heart must arrive at the left chamber, but there is no direct pathway between them. The thick septum of the heart is not perforated and does not have visible pores as some people thought or invisible pores as Galen thought. The blood from the right chamber must flow through the vena arteriosa (*[*pulmonary artery*]*) to the lungs, spread through its substances, be mingled there with air, pass through the arteria venosa (*[*pulmonary vein*]*) to reach the left chamber of the heart"*

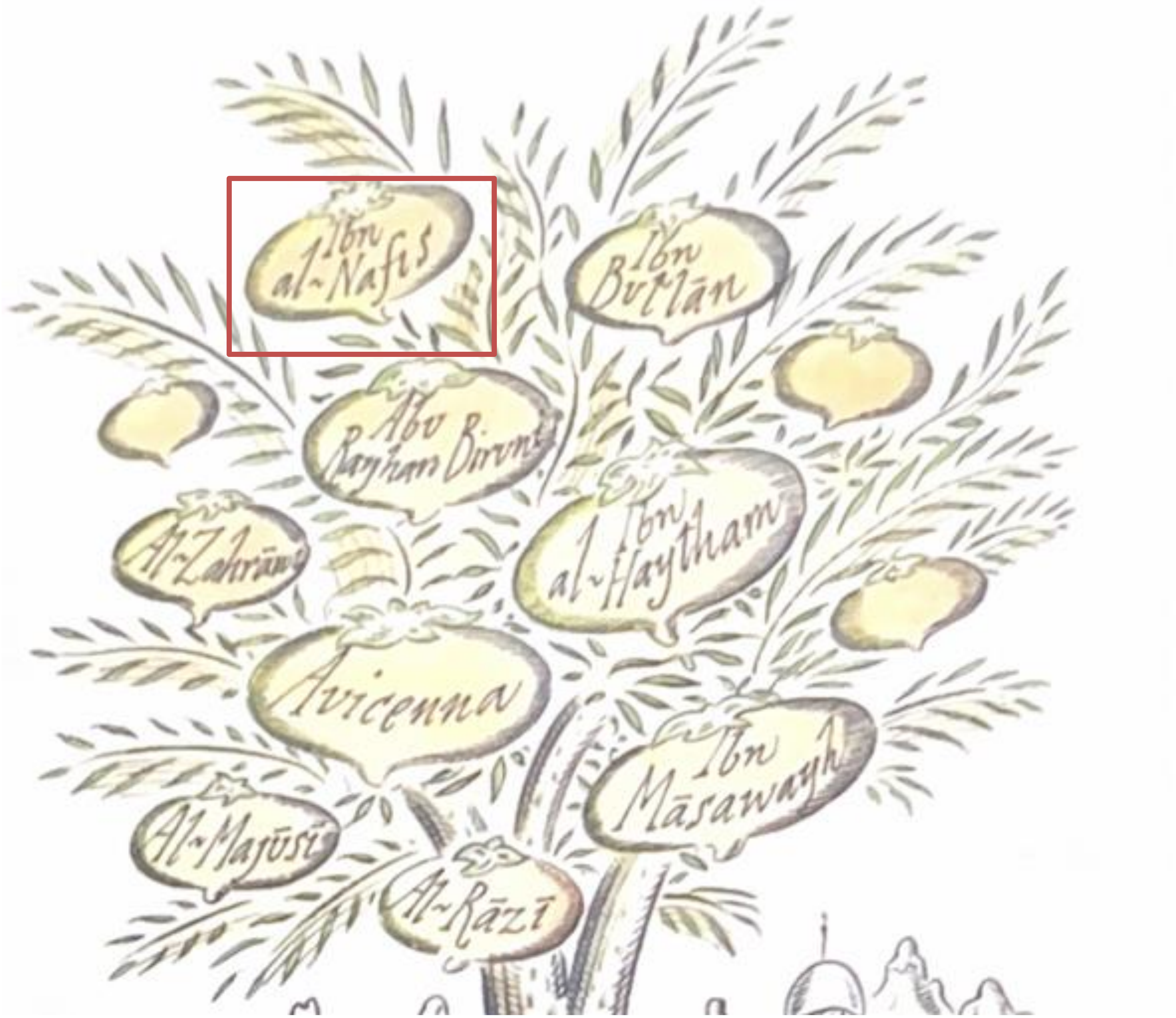

Ibn Al Haytham

- Book of optics in 1011 first described the passage of light reflected off an object into the eye where visual perception occurs in the brain.
- He was first to state that the incident ray, the reflected ray, and the normal to the surface all lie in a same plane perpendicular to reflecting plane.

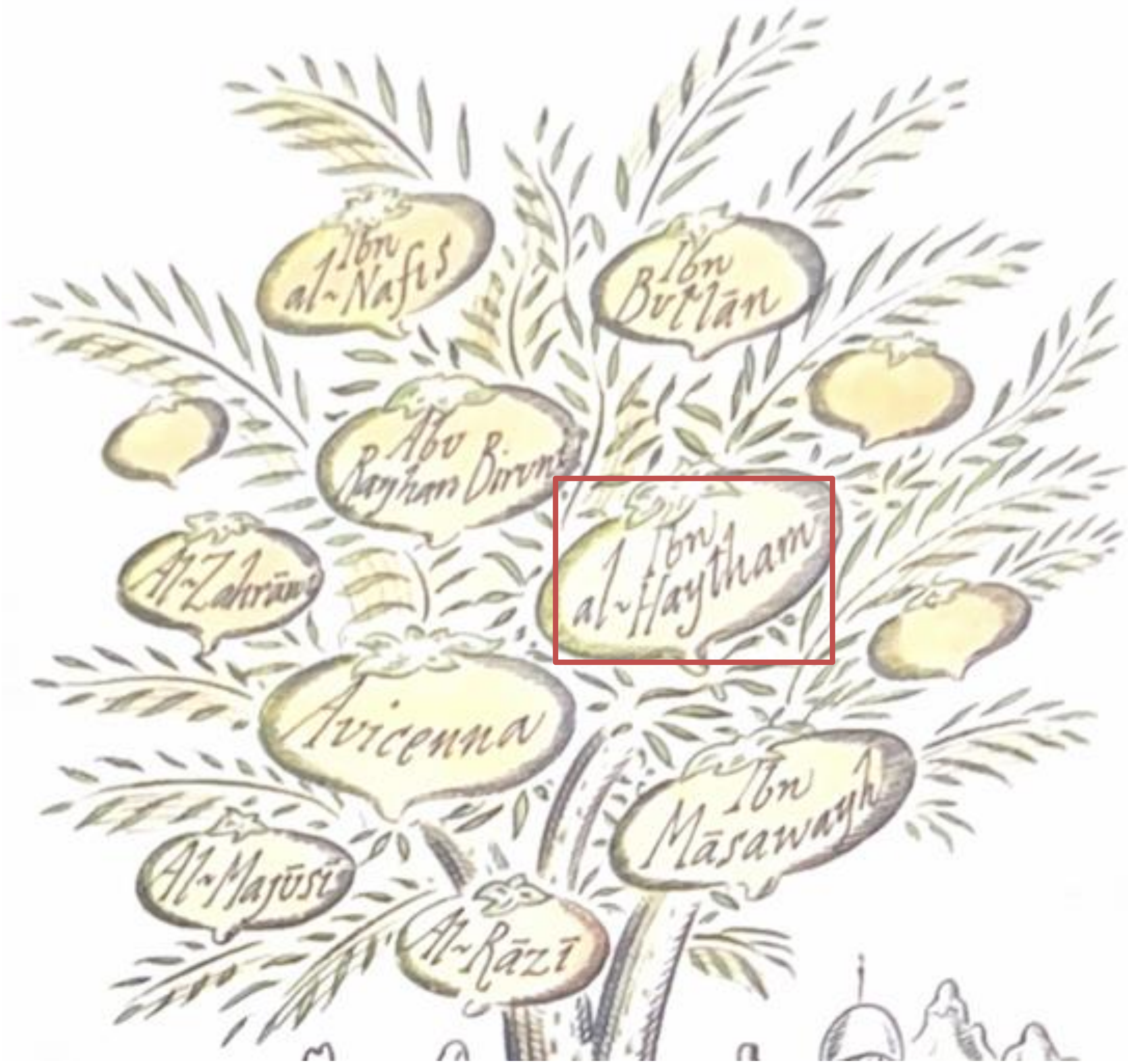

Ibn Sina or Avicenna

- Wrote and compiled the Canon of Medicine, which summised all medical knowledge at the time, 1025

- William Osler described the *Canon* as "the most famous medical textbook ever written," noting that it remained "a medical bible for a longer time than any other work."

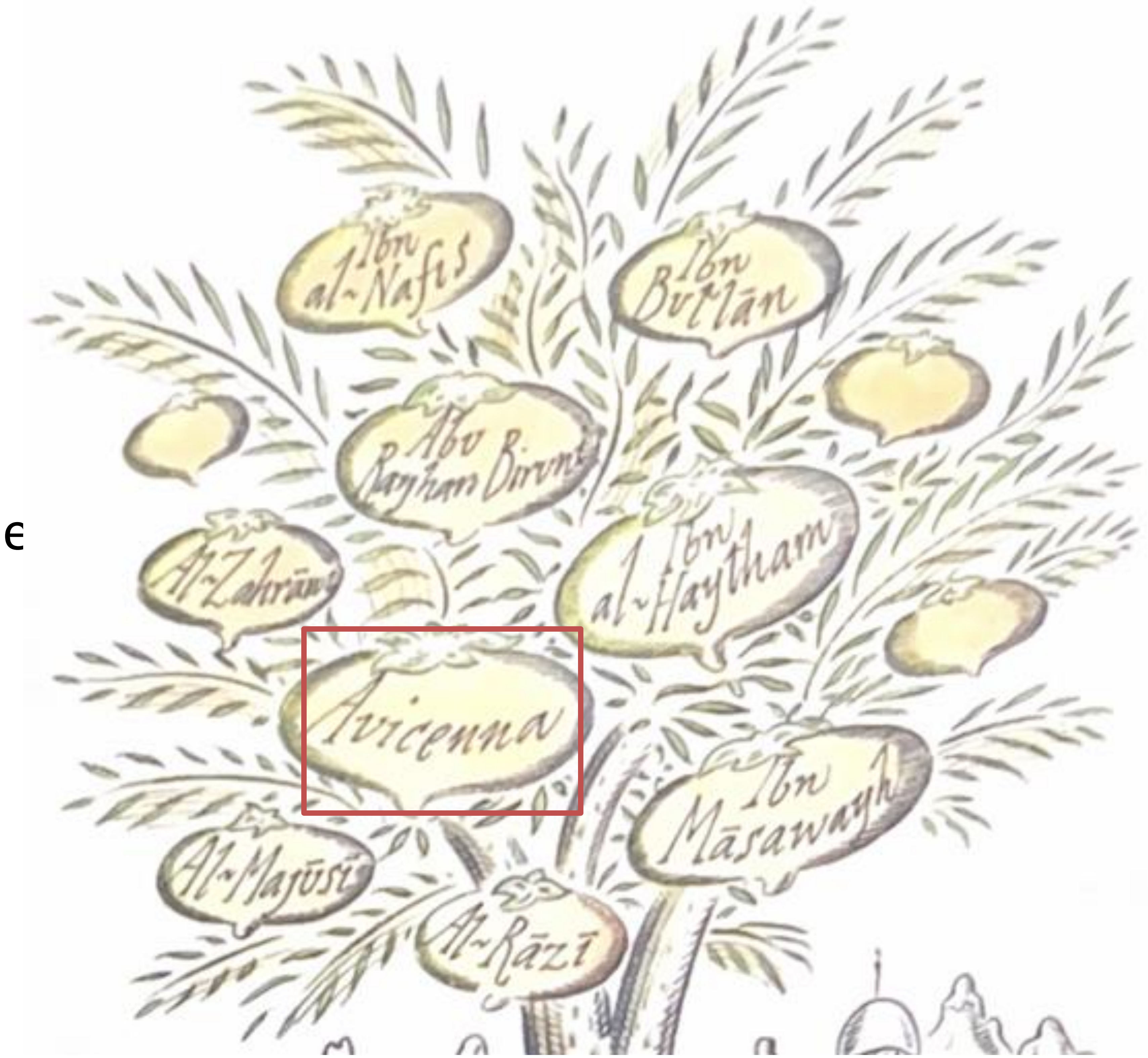

Al-Zahrawi or Abulcassis

- Surgical pioneer in the 900s exploring ophthalmology, obstetrics, orthopaedics and neurosurgery, urology inventing >200 instruments.
- Developed a intraurethral device to crush bladder stone without excision
- Described the hereditary nature of haemophilia
- First to use absorbable sutures (catgut)
- Describes the evacuation of superficial intracranial fluid in hydrocephalic children.
- Advocated the treatment of patients despite their social status

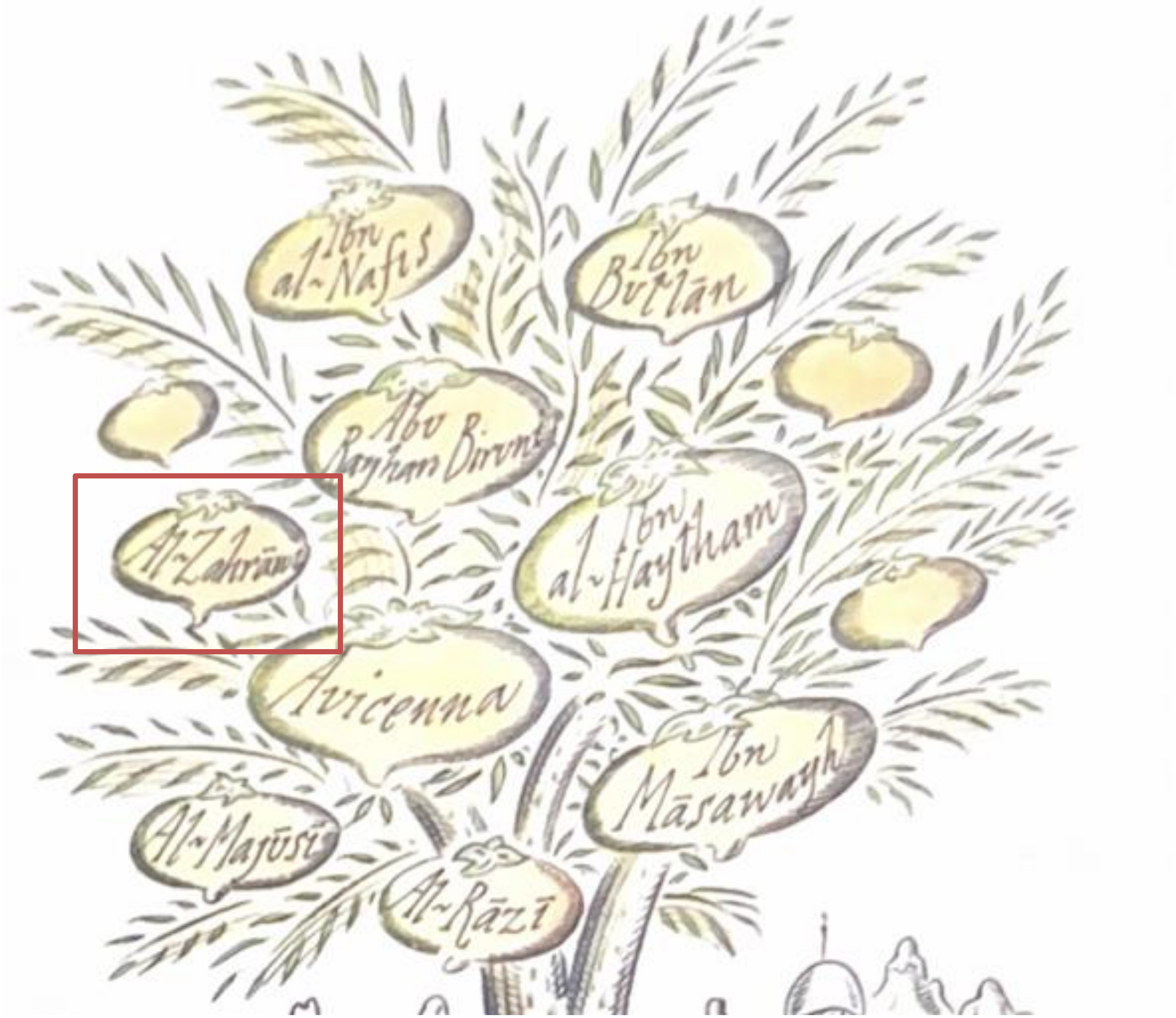

Al-Zahrawi or Abulcassis

- On the importance of training;

"Before practising surgery one should gain knowledge of anatomy and the function of organs so that they will understand their shape, connections and borders. They should become thoroughly familiar with nerves muscles bones arteries, and veins. If one does not comprehend the anatomy and physiology one can commit a mistake which will result in the death of the patient. I have seen someone incise into a swelling in the neck thinking it was an abscess, when it was an aneurysm and the patient dying on the spot."

Al Zahrawi, d. 1013

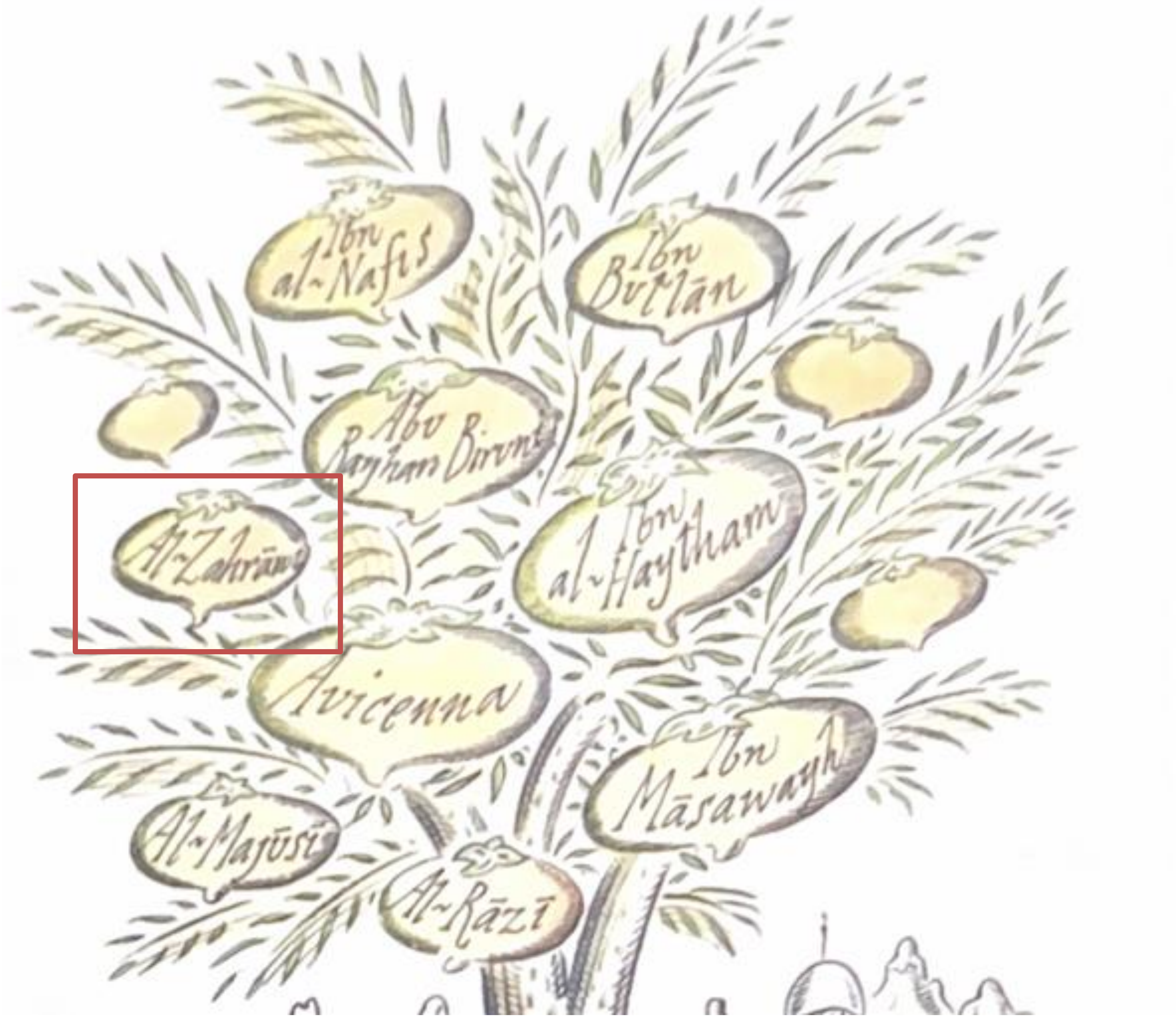

Al-Zahrawi or Abulcassis

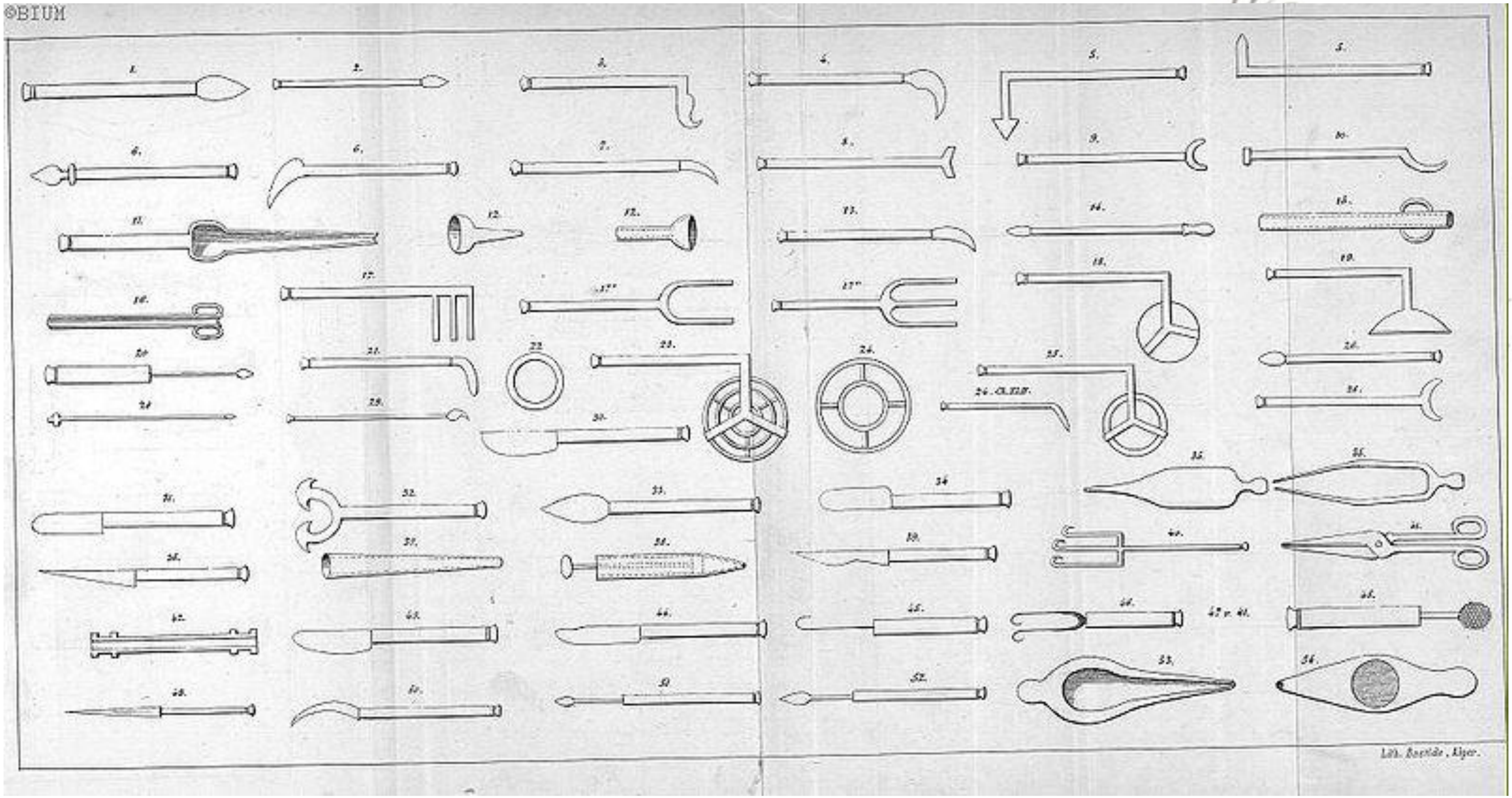

Supplement: Supplementary file 1 [file Data_Sheet_1.pdf]
